# A GM-CSF-neuroantigen tolerogenic vaccine elicits inefficient antigen recognition events below the CD40L triggering threshold to expand CD4^+^ CD25^+^ FOXP3^+^ Tregs that inhibit experimental autoimmune encephalomyelitis (EAE)

**DOI:** 10.1186/s12974-020-01856-8

**Published:** 2020-06-10

**Authors:** Cody D. Moorman, Alexander G. Bastian, Kayla B. DeOca, Mark D. Mannie

**Affiliations:** Department of Microbiology and Immunology, Brody School of Medicine, East Carolina University, Greenville, NC 27834 USA

**Keywords:** FOXP3^+^ Tregs, Immune tolerance, Tolerogenic vaccine, EAE/MS, Neuroimmunology, GM-CSF, Neuroantigen

## Abstract

**Background:**

Tolerogenic vaccines represent antigen-specific interventions designed to re-establish self-tolerance and thereby alleviate autoimmune diseases, which collectively comprise over 100 chronic inflammatory diseases afflicting more than 20 million Americans. Tolerogenic vaccines comprised of single-chain GM-CSF-neuroantigen (GMCSF-NAg) fusion proteins were shown in previous studies to prevent and reverse disease in multiple rodent models of experimental autoimmune encephalomyelitis (EAE) by a mechanism contingent upon the function of CD4^+^ CD25^+^ FOXP3^+^ regulatory T cells (Tregs). GMCSF-NAg vaccines inhibited EAE in both quiescent and inflammatory environments in association with low-efficiency T cell receptor (TCR) signaling events that elicited clonal expansion of immunosuppressive Tregs.

**Methods:**

This study focused on two vaccines, including GMCSF-MOG (myelin oligodendrocyte glycoprotein 35–55/MOG^35–55^) and GMCSF-NFM (neurofilament medium peptide 13–37/NFM^13–37^), that engaged the transgenic 2D2 TCR with either low or high efficiencies, respectively. 2D2 mice were crossed with FOXP3 IRES eGFP (FIG) mice to track Tregs and further crossed with *Rag*^*−/−*^ mice to reduce pre-existing Treg populations.

**Results:**

This study provided evidence that low and high efficiency TCR interactions were integrated via CD40L expression levels to control the Treg/Tcon balance. The high-efficiency GMCSF-NFM vaccine elicited memory Tcon responses in association with activation of the CD40L costimulatory system. Conversely, the low-efficiency GMCSF-MOG vaccine lacked adequate TCR signal strength to elicit CD40L expression and instead elicited Tregs by a mechanism that was impaired by a CD40 agonist. When combined, the low- and high-efficiency GMCSF-NAg vaccines resulted in a balanced outcome and elicited both Tregs and Tcon responses without the predominance of a dominant immunogenic Tcon response. Aside from Treg expansion in 2D2-FIG mice, GMCSF-MOG caused a sustained decrease in TCR-β, CD3, and CD62L expression and a sustained increase in CD44 expression in Tcon subsets. Subcutaneous administration of GMCSF-MOG without adjuvants inhibited EAE in wildtype mice, which had a replete Treg repertoire, but was pathogenic rather than tolerogenic in 2D2-FIG-*Rag1*^*−/−*^ mice, which lacked pre-existing Tregs.

**Conclusions:**

This study provided evidence that the GMCSF-MOG vaccine elicited antigenic responses beneath the CD40L triggering threshold, which defined an antigenic niche that drove dominant expansion of tolerogenic myelin-specific Tregs that inhibited EAE.

## Background

Multiple sclerosis (MS) is a debilitating autoimmune disease of the central nervous system (CNS) and is the leading cause of non-traumatic disability in young adults. MS is driven by genetic and environmental factors that result in inflammatory lesions in white and grey matter of the CNS, demyelination, axonal damage, neuronal death, and CNS atrophy [1]. Currently, FDA-approved therapies for MS include first-line therapies, which are immunomodulatory and have modest efficacy, and second-line therapies which are efficacious but broadly immunosuppressive. The clinical efficacy of a MS-specific therapy directly correlates with the level of immunosuppression and with adverse risk for opportunistic infection and cancer [2, 3]. Early diagnosis and therapeutic intervention may delay the short-term progression (2–3 years) but generally has marginal impact on long-term (> 3 years) disease progression [4]. The main shortcoming is that current MS therapies are broad nonspecific immunosuppressants that lack disease-specific activity. Ideal therapies would target pathogenic clonotypes without impairment of the T and B cell repertoires needed for host defense [5].

Tolerogenic vaccines present a solution to this treatment conundrum. Tolerogenic vaccines represent a new treatment paradigm for MS in that these interventions combine MS-disease specificity with the potential for robust efficacy. Tolerogenic vaccines are antigen-specific therapies designed to restore self-tolerance without the induction of general immunosuppression [6]. Because MS is a T cell-driven disease, tolerogenic vaccines are designed to target pathogenic myelin-reactive CD4^+^ T cell responses through the induction of Tregs [5, 6]. Although many tolerogenic vaccine platforms are currently in development, none have received FDA approval for the treatment of autoimmunity [7]. Therefore, vaccine platforms are needed that induce robust tolerance and are efficacious in inflammatory environments. Vaccine strategies designed to expand memory/effector FOXP3^+^ Tregs are ideal because such vaccines would provide long-lasting suppressive memory by reinforcing CNS-specific mechanisms of active dominant tolerance [8].

FOXP3^+^ Tregs are conducive vaccine targets because this regulatory subset mediates self-tolerance and prevents autoimmunity. The master lineage-specific transcription factor FOXP3 phenotypically defines the Treg lineage, and genetic deficiency of FOXP3 confers a lack of Tregs and a fatal autoimmune disease known as immunodysregulation polyendocrinopathy enteropathy X-linked in humans and scurfy in mice [9, 10]. FOXP3^+^ Tregs mediate disease resistance and recovery from experimental autoimmune encephalomyelitis (EAE), an animal model of MS [11–15]. Treg numbers, suppressive capacity, and migratory abilities are also reduced in MS patients compared to healthy controls [15, 16]. Given that Tregs are deficient in autoimmune disease, a major challenge is to devise tolerogenic vaccines that restore tolerance to tissue-specific self-antigens as a solution for chronic inflammation and autoimmunity.

Single-chain fusion proteins comprised of a N-terminal GM-CSF domain and a C-terminal immunodominant neuroantigen (NAg) domain represent a tolerogenic vaccine platform that fulfills many of the criteria needed for clinical translation. These GMCSF-NAg vaccines were robust tolerogens in multiple rodent models of EAE [6, 17–21]. GMCSF-NAg fusion proteins targeted the tethered NAg for enhanced presentation on major histocompatibility complex class II (MHCII) glycoproteins by myeloid antigen presenting cells (APCs). For example, rat GM-CSF-myelin basic protein peptide 69–87 (GMCSF-MBP) targeted MBP to myeloid APCs to confer an approximate 1000-fold increase in antigen potency [17]. This vaccine-based mechanism of antigenic targeting was blocked by free GM-CSF in vitro. GMCSF-NAg fusion proteins, including rat GMCSF-MBP^69–87^, mouse GMCSF-MOG^35–55^, and mouse GMCSF-proteolipid protein (PLP)^139–151^ peptide were effective therapeutic and prophylactic treatments in the respective models of EAE, including the monophasic Lewis rat model [17], the chronic C57BL/6 model [18, 20], and the relapsing-remitting SJL model of EAE [18, 20]. Thus, these findings reveal close associations among GM-CSF conditioning of myeloid APCs, GM-CSF targeting of ‘self’ neuroantigens for presentation by those GM-CSF-conditioned APCs, and tolerance induction in multiple models of EAE.

The tolerogenic effects of GMCSF-NAg were dependent on vaccine-induced Tregs because ablation of Tregs following GMCSF-MOG vaccination abrogated vaccine-induced tolerance [21]. The ability of GMCSF-NAg to induce Tregs was investigated by use of 2D2-FIG mice which have a GFP reporter immediately downstream of FOXP3 expression and a transgenic TCR that recognizes MOG^35–55^ as a low-affinity antigen and NFM^13–37^ as a high-affinity antigen. A single subcutaneous (SC) vaccination of 2D2-FIG mice with GMCSF-MOG in saline, the low-affinity vaccine, led to systemic Treg induction in the blood, spleen, and lymph nodes which appeared 3 days post-vaccination and persisted for several weeks. In contrast, vaccination of 2D2-FIG mice with the high-affinity GMCSF-NFM vaccine did not result in Treg induction. Therefore, the quality of the antigen recognition was a major parameter defining the outcome of GMCSF-NAg-based vaccination [21]. The GMCSF-NAg platform was unique among tolerogenic vaccines because GMCSF-NAg induced tolerance and elicited NAg-specific Tregs even when administered in inflammatory environments [20]. That is, GMCSF-MOG prevented severe EAE in C57BL/6 mice when injected adjacent to the MOG^35–55^/complete Freund’s adjuvant (CFA) emulsion as well as when directly emulsified in the MOG^35–55^/CFA emulsion. The GMCSF-MOG vaccine also elicited a robust systemic Treg response even when administered in strong immunogenic adjuvants such as CFA and aluminum hydroxide gel [21].

These prior studies however left a major unresolved question regarding the nature of the antigen recognition events that connected the GMCSF-NAg vaccine/Treg axis with tolerogenic outcomes. This study addresses this gap in knowledge by showing that the strength of the GMCSF-NAg antigenic domain was translated from T cells to the APC via the CD40L/CD40 costimulatory pathway as a critical node ultimately controlling the Treg/Tcon balance. This observation was congruent with the concept that CD40L serves as a critical gateway for adaptive immunity. Most notably, deficiencies in *CD40L* cause Hyper-IgM Syndrome, which manifests as profound deficits in cell-mediated and humoral immunity, together with class switching defects marked by accumulation of IgM antibodies and a paucity of downstream isotypes such as IgG and IgA [22]. Furthermore, genetic *Cd40lg* deficiency or antibody-mediated CD40L blockade inhibits EAE in mice [23–27]. CD40L mediates “T cell help” in that agonistic ligation of the TCR triggers expression of CD40L on activated CD4^+^ T cells that then interacts with CD40 on B cells, macrophages, dendritic cells (DCs) to elicit APC activation and induction of B7 costimulatory molecules to drive immunogenic responses. This study advances this concept by providing evidence that the efficiency of TCR antigen recognition is translated via CD40L expression to set thresholds gating tolerogenic and immunogenic immunity, with self-antigens generally below and foreign antigens generally above CD40L induction thresholds to set the balance of Treg-mediated tolerance versus Tcon-mediated immunogenicity.

## Methods

### Mice

C57BL/6J (000664), B6.SJL-Ptprca Pepcb/BoyJ (CD45.1 002014), B6.Cg-*Foxp3*^*tm*2*Tch*^/J (FIG Foxp3-IRES-GFP 006772), B6.129S7-*Rag1*^*tm*1*Mom*^/J (*Rag1*^−/−^ 002216), and C57BL/6-Tg(Tcra2D2,Tcrb2D2)1Kuch/J (2D2 MOG^35–55^-specific TCR transgenic 006912) mouse strains were obtained from the Jackson Laboratory (Bar Harbor, ME) and were maintained as a colony in the Department of Comparative Medicine at East Carolina University. 2D2-FIG, CD45.1-2D2-FIG, and 2D2-FIG-*Rag1*^−/−^ mice were obtained through intercross breeding and were routinely screened for CD45.1/CD45.2 alleles as well as Vβ11 and Vα3.2 MOG-specific TCR by flow cytometric analysis. GFP expression from FIG mice was used as a surrogate marker of FOXP3 expression. Animal care and use were performed in accordance with approved animal use protocols and guidelines of the East Carolina University Institutional Animal Care and Use Committee.

### Reagents and recombinant proteins

The synthetic peptides MOG^35–55^ (MEVGWYRSPFSRVVHLYRNGK) and NFM^13–37^ (RRVTETRSSFSRVSGSPSSGFRSQS) were obtained from Genscript (Piscataway, NJ). Monoclonal antibodies FGK4.5 (rat anti-mouse CD40 IgG2a) and 2A3 (rat anti-trinitrophenol IgG2a) were purchased from BioXcell (West Lebanon, NH). Derivation, expression, purification, and bioassay of the murine GM-CSF, GMCSF-MOG (peptide MOG^35–55^), GMCSF-NFM (peptide NFM^13–37^), and GMCSF-OVA (peptide Ovalbumin (OVA) ^323–339^) fusion proteins were described in previous studies [18, 19, 21]. These fusion proteins were expressed in stably transfected human embryonic kidney (HEK) cells and Chinese hamster ovary (CHO) cells. The fusion proteins were purified by incubation of expression supernatants with Ni-NTA Agarose beads followed by extensive washing of the affinity chromatography columns with escalating concentrations of imidazole (50 mM NaH_2_PO_4_, 500 mM NaCl, and either 10, 20, or 60 mM imidazole, pH 8.0). Recombinant proteins were eluted with 250 mM imidazole (pH 8.0) and were concentrated and diafiltrated in Amicon Ultra-15 centrifugal filter devices (EMD Millipore, Billerica, MA). Protein quantity was assessed by absorbance at 280 nm, and purity was assessed by SDS-PAGE. Bioactivity of the GM-CSF domain was assayed by proliferation of bone marrow cells, and the bioactivity of the antigenic domain was assayed by proliferation of 2D2 T cells and OTII T cells in the presence of irradiated DCs. Recombinant rat TGF-β1 was expressed by use of transfected HEK cells and purified as previously described [14]. Recombinant rat IL-2 was derived from a baculovirus expression system [28]. The PC61-5.3 anti-CD25 rat IgG1 (λ) hybridoma was obtained from ATCC, and the PC61 mAb was purified as previously described [13].

### Flow cytometric analyses of leukocytes

For vaccination studies and ex vivo/in vitro analyses, mice were vaccinated or cells were stimulated with designated antigens (e.g., GMCSF-MOG, GMCSF-NFM, MOG^35–55^, NFM^13–37^) and peripheral blood mononuclear cells (PBMC) were collected via the submandibular vein and were diluted into sodium citrate (130 mM). Alternatively, lymph nodes and spleens were dissected from mice and pressed through 70 μm cell strainer (Corning, NY) to obtain a single cell suspension. Cells were washed with Hank’s buffered saline solution with 2% heat-inactivated fetal bovine serum, and cells were stained with designated cocktails of fluorochrome-conjugated antibodies for 1 h in the dark at 4 °C. Erythrocytes were lysed by incubating samples on ice with 3 ml of ammonium chloride lysis buffer (150 mM NH_4_Cl, 10 mM NaHCO_3_, 1.2 mM EDTA-pH 7.2). FITC-, PE-, or APC-conjugated EasyComp fluorescent particles (3.0–3.4 μm, Spherotech, Lake Forest, IL) were added to samples in phosphate-buffered saline (PBS) to calculate the absolute number of cells. Samples were run on a Becton-Dickson LSRII flow cytometer with FACSDiva software (San Jose, CA), and results were analyzed with FlowJo software (Ashland, OR). Fluorochrome-conjugated mAbs (BioLegend, San Diego, CA) included CD3-BV421/CD3-PE-Dazzle 594 (17A2 or 145-2C11), CD4-BV785 (GK1.5), CD25-BV421/CD25-APC (PC61), CD44-BV421 (IM7), CD40-PE (MR1), CD45.1-APC/CD45.1-BV421 (A20), CD45.2-APC (104), CD62L-APC (MEL-14), Trinitrophenol-KLH-PE (HTK888), TCR-Vα3.2-PE (RR3-16), and TCR-Vβ11-PE/Vβ11-AF647 (KT11). To stain CD40L, T cells were stimulated in the presence or absence of MOG^35–55^, NFM^13–37^, GMCSF-MOG, GMCSF-NFM, or GM-CSF for 4 h and subsequently incubated with 10 μg/mL anti-mouse CD40L-PE (Armenian Hamster IgG, MR1) or the isotype control anti-mouse Trinitrophenol-KLH-PE (Armenian Hamster IgG, HTK888) for an additional 2 h at 37 °C.

### In vitro assays

To determine if vaccine-induced Tregs were suppressive, 2D2-FIG mice were given a SC vaccination of 4 nmol GMCSF-MOG in saline to induce Tregs. On day 7, CD4^+^ GFP^high^ (FOXP3^high^) Tregs were sorted from the vaccinated splenocytes by use of a Becton-Dickson FACSAria^TM^ Fusion flow cytometer (San Jose, CA). T cell responders were isolated from a naïve 2D2-FIG mouse by use of an untouched CD4^+^ MACS system (Miltenyi Biotech, Bergisch Gladbach, Germany). Tregs were mixed with T responders and activated with 100,000 irradiated C57BL/6 splenocytes and 1 μM MOG^35–55^ in complete RPMI (10% heat-inactivated fetal bovine serum, 2 mM glutamine, 100 μg/ml streptomycin, 100 U/ml penicillin, 50 μM 2-mercaptoethanol).

For antigen-recall assays, 2D2-FIG mice were vaccinated with GMCSF-MOG, GMCSF-NFM, or saline. Splenic CD4^+^ T cells were isolated by use of an anti-CD4 (L3T4) MACS system (Miltenyi Biotech, Bergisch Gladbach, Germany). Purified CD4^+^ T cells (25,000) from vaccinated mice were activated with 200,000 irradiated naïve C57BL/6 splenocytes and 1 nM–10 μM of MOG^35–55^ or NFM^13–37^ in complete RPMI.

For antigen targeting assays, short-term C57BL/6 DC lines were generated by a 4-day culture in GM-CSF followed by a 2-day culture in GM-CSF and IL-4. These DCs (20,000 cells/well) were then cultured with 32 nM–3.2 μM of MOG^35–55^, GMCSF-MOG, GM-CSF + MOG^35–55^, NFM^13–37^, GMCSF-NFM, or GM-CSF + NFM^13–37^ for 2 days. DCs were washed extensively and were parked in a subsequent 4-day culture without antigen or cytokines. The DCs were then irradiated and were cultured for 3 days with purified splenic CD4^+^ responder 2D2-FIG T cells (20,000 cells/well) that were isolated by use of an anti-CD4 (L3T4) MACS system (Miltenyi Biotech, Bergisch Gladbach, Germany). No antigen was added to this final 3-day culture. In this system, antigens acquired by DCs in the initial 2-day culture and retained during the subsequent 4-day culture were presented to T cell responders in the final 3-day culture.

For the IL-2 production assays, naïve 2D2-FIG splenocytes were cultured with 1 μM MOG or 100 nM NFM in complete RPMI. These concentrations represented the optimal concentrations for stimulation of peak responses. At indicated time points (*x*-axis), culture supernatants were harvested and stored at – 20 °C. To test IL-2 bioactivity, supernatants that were collected at the designated time-points and were used to stimulate a transformed, IL-2-dependent mouse T cell line (SJL-PLP.1) at 20,000 cells/well.

For the in vitro assays enumerated in this section, cultures were pulsed with 1 μCi [^3^H]thymidine (6.7 Ci/mmol, New England Nuclear, Perkin Elmer, Waltham, MA, USA) during the last 24 h of a 72-h culture. Cultures were harvested onto filters by use of a Tomtec Mach III harvester (Hamden, CT, USA). [^3^H]thymidine incorporation into DNA was measured by use of a Perkin Elmer MicroBeta2 liquid scintillation counter.

### Generation and maintenance of Treg and Tcon lines

To generate Treg and Tcon lines, splenocytes from congenic CD45.1 2D2-FIG mice were FACS-sorted into GFP^+^ (Tregs) and GFP^-^ (Tcon) CD3^+^ CD4^+^ T cell subsets and cultured overnight. The sorted Tregs and Tcons were activated at 4 × 10^5^ cells/ml for 3 days with 2.5 μg/ ml Con-A, IL-2, and irradiated DCs (10^5^/ml) in the presence (Tregs) or absence (Tcons) of 1 nM TGF-β. The Treg and Tcon lines were then propagated with IL-2, and Tregs were also cultured for 6 days with the anti-CD25 mAb PC61 to maintain Treg stability as previously described [29]. Tregs were extensively washed to remove PC61 and propagated an additional 3 days in IL-2. Following a total of 13 days in culture, the T cells were adoptively transferred to congenic CD45.2 2D2-FIG-*Rag1*^*−/−*^ mice.

### Induction and assessment of EAE

To induce EAE in C57BL/6 mice, CFA (Incomplete Freund's Adjuvant with 4 mg/ml heat-killed mycobacterium tuberculosis H37Ra, BD Biosciences, Franklin Lakes, NJ) was mixed 1:1 with MOG^35–55^ in PBS. The CFA/antigen mixture was emulsified by sonication. EAE was elicited by injection of 200 μg MOG^35–55^ in a total volume of 100 μl emulsion via three SC injections of 33 μl across the lower back. Each mouse received separate intraperitoneal injections (400 ng i.p.) of Pertussis toxin (EMD Millipore, Billerica, MA) in PBS on days 0 and 2. To induce EAE in the 2D2-FIG-*Rag1*^*−/−*^ mice, GMCSF-MOG or GMCSF-NFM (4 nmol) was administered SC in saline in two locations (100 μL each) on the hind back, in the absence of any adjuvant. All immunizations were performed under isoflurane anesthesia (Abbott Laboratories, Chicago, IL). Mice were assessed daily for clinical score and body weight. The following scale was used to score the clinical signs of EAE: 0, no disease; 0.5, partial paralysis of tail without ataxia; 1.0, flaccid paralysis of tail or ataxia but not both; 2.0, flaccid paralysis of tail with ataxia or impaired righting reflex; 3.0, partial hind limb paralysis marked by inability to walk upright but with ambulatory rhythm in both legs or impaired reflexes associated with foot clasping or disequilibrium with head tilt; 3.5, same as above but with full paralysis of one leg; 4.0, full hindlimb paralysis; 5.0, total hindlimb paralysis with forelimb involvement or moribund. A score of 5.0 was designated a humane endpoint.

### EAE analysis and statistics

Incidence of EAE was the number of EAE-afflicted mice divided by the total group size. Maximal scores were calculated as the most severe EAE score for each mouse. Mice that did not exhibit EAE had a score of 0, and these scores were included in the group average. Mice that exhibited humane endpoints as assessed by body weight loss, body score, or clinical score of 5.0 were subjected to humane euthanasia and were omitted from scoring thereafter. Time-course graphs portrayed daily mean maximal scores. Cumulative and maximal EAE scores were analyzed by either a Mann-Whitney *U* test (two groups) or the Kruskal-Wallis test (greater than two groups). To calculate percent maximal weight loss, 100% body weight was assigned as the maximal body weight obtained on day 0 and daily body weights were calculated for each day after normalization to the day 0 value. The minimum body weight was defined as the lowest body weight from day 1 until the end of the experiment. Maximal weight loss was calculated by subtraction of the normalized minimum value from the day 0 value. Negative weight loss values represented weight gain. Weight loss was analyzed by two-tailed Student’s *t* test. Mean EAE and weight loss data were shown with the standard error of the mean (SEM). Kruskal-Wallis test significance values were adjusted by the Bonferroni correction for multiple tests. A two-way repeated measures ANOVA was used to determine daily statistical significance. A *p* value ≤ 0.05 was considered significant. Error bars represent SEM unless designated otherwise.

## Results

In this study, we did not attempt to distinguish thymic (tTregs) (i.e., also referred to as natural Tregs or nTregs) that arise during thymic ontogeny [30, 31] from peripheral Tregs (pTregs) that differentiate de novo from naïve T cell precursors in peripheral tissues upon recognition of tissue-specific self-antigens or foreign environmental antigens. We also used iTregs, which are Tregs induced from naïve precursors during culture with a TCR agonist and TGF-β. Rather, we used the encompassing term of pre-existing Tregs simply because the field currently lacks validated markers that distinguish the tTreg, pTreg, and iTreg subsets. We refer to antigen efficiencies (i.e., low versus high) as an encompassing term for the ability of an antigen to elicit the productive activation of a T cell clonotype rather than more specific terms of TCR-antigen potency, efficacy, affinity, or avidity simply to accommodate uncertainties in the field regarding TCR signaling modalities.

### High-efficiency antigens but not low-efficiency antigens elicited CD40L in 2D2-FIG splenocytes

**Fig. 1:**
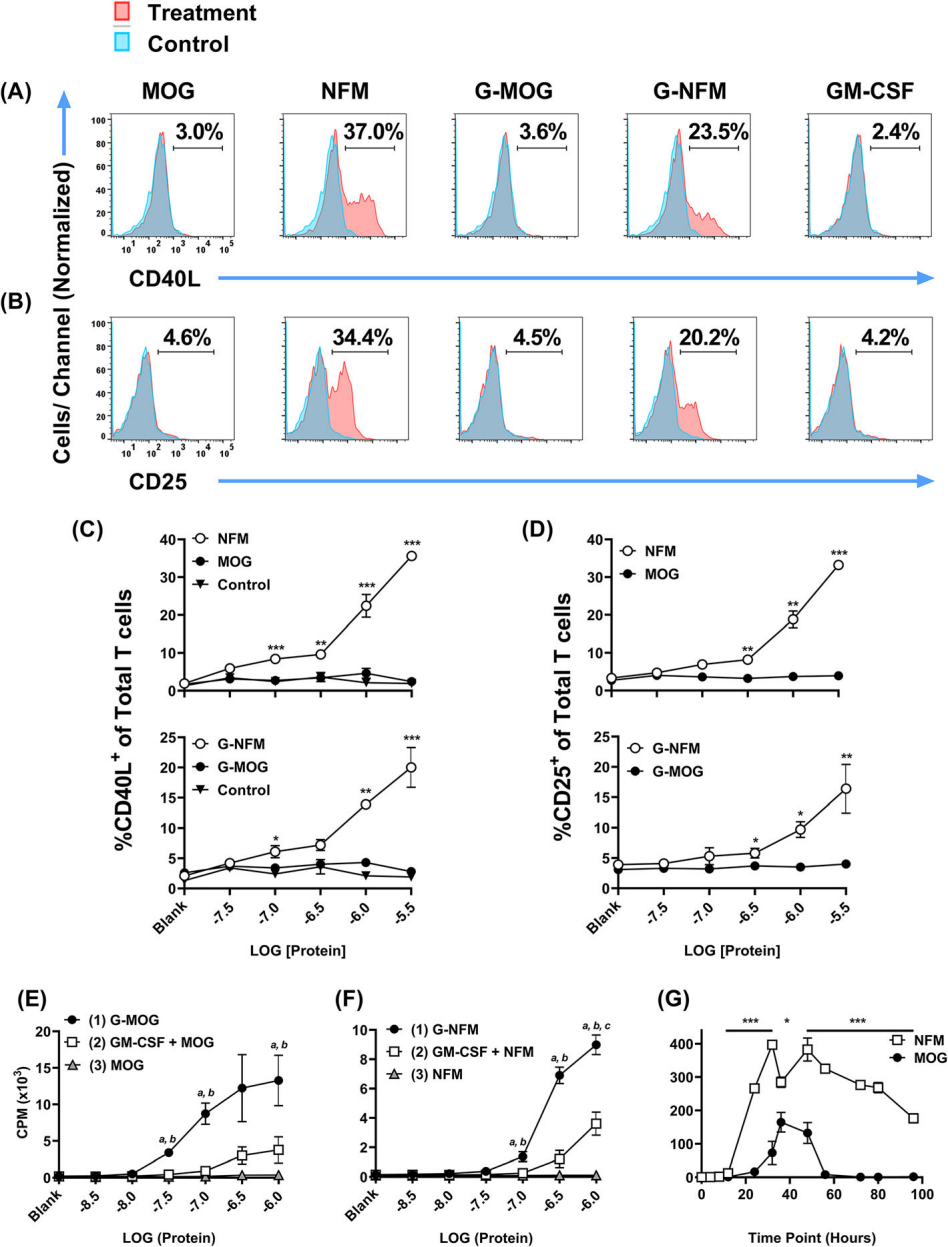
High-efficiency antigens but not low-efficiency antigens elicited CD40L in 2D2-FIG splenocytes. 2D2-FIG splenocytes were cultured with 3.2 μM MOG^35–55^ (MOG), NFM^13–37^ (NFM), GMCSF-MOG (G-MOG), GMCSF-NFM (G-NFM), or GM-CSF in duplicate. After 4 h of culture, 10 μg/ ml PE-conjugated anti-mouse CD40L mAb (Armenian Hamster IgG, MR-1) or control PE-conjugated anti-trinitrophenol-KLH mAb (Armenian Hamster IgG, HTK888) were added to cultures and incubated for an additional 2 h. Cells were then stained for CD3, CD4, and CD25. Shown are representative histograms of CD40L (**a**) and CD25 (**b**) expression (*x*-axis) of CD3^+^ CD4^+^ T cells activated with designated antigens or cytokines. Shown are percentages of CD40L^+^ (**c**) or CD25^+^ T cells (**d**) of the total CD3^+^ CD4^+^ T cell population following culture with designated treatments. C57BL/6 DCs (20,000/well) were cultured for 2 days with designated concentrations of MOG, G-MOG, or GM-CSF + MOG (**e**), or NFM, G-NFM, or GM-CSF + NFM (**f**). DCs were washed extensively and then were parked for 4 days in a second culture without antigen. DCs were then irradiated and cultured in the absence of antigen with naïve responder 2D2-FIG T cells (25,000/well). Thus, antigen acquired during the first culture that persisted over the second culture was assayed by responder T cells in the third culture. **g** 2D2-FIG splenocytes were cultured with 1 μM MOG or 100 nM NFM in triplicate. At indicated time points (*x*-axis), culture supernatants were harvested and assayed for IL-2 bioactivity by use of a transformed, IL-2-dependent mouse T cell line (SJL-PLP.1, 20,000 cells/well). **e**–**g** Cultures were pulsed with 1 μCi of [^3^H]thymidine during the last 24 h of a 3-day culture. These data are representative of three independent experiments. **c, e**, **f** Statistical significance was analyzed by use of a one-way ANOVA. **d**, **g** Statistical significance was analyzed by use of a one-tailed *t* test. (*p < 0.05, **p < 0.01, ***p < 0.001). **e** (*a*) G-MOG vs GM-CSF + MOG (*p < 0.05*) and (*b*) G-MOG vs MOG (*p < 0.05). ***f** (*a*) G-NFM vs GM-CSF + NFM (*p < 0.05*) and (*b*) G-NFM vs NFM and (*c*) GM-CSF + NFM vs NFM (*p < 0.05*). Error bars represent standard deviations

Given that the low-efficiency GMCSF-MOG vaccine favored Treg induction whereas the high-efficiency GMCSF-NFM vaccine elicited dominant Tcon responses [21], we hypothesized that TCR engagement efficiency may control the CD40L/CD40 co-stimulatory pathway as a primary intermediate controlling the Treg/Tcon balance. Thus, we compared high-efficiency antigens (NFM^13–37^ or GMCSF-NFM) with low-efficiency antigens (MOG^35–55^ and GMCSF-MOG) in 2D2-FIG splenocyte cultures for induction of CD40L. CD25 was assessed as a surrogate activation marker (Fig. 1). Consistent with this hypothesis, NFM^13–37^ and GMCSF-NFM (1.0–3.2 μM) elicited high percentages of CD40L^+^ T cells (Fig. 1a, c) and CD25^+^ T cells (Fig. 1b, d) compared to T cells stimulated with GM-CSF or stained with an isotype control. In contrast, MOG^35–55^ and GMCSF-MOG did not elicit detectable levels of CD40L or CD25. These findings indicated that TCR recognition efficiency sets the activation threshold for the CD40L/CD40 pathway.

Despite the lack of activity in CD40L induction assays, GMCSF-MOG had superior activity in antigenic targeting assays (Fig. 1e). DCs were cultured with GMCSF-MOG, GM-CSF+ MOG^35–55^, or MOG^35–55^ alone in an initial 2-day culture, were extensively washed, parked in a subsequent 4-day culture without antigen, and then assayed in a 3rd culture, again without antigen, for presentation of antigens that were acquired during the first culture. Targeting activity was evident in that GMCSF-MOG was more stimulatory than the mixture of GM-CSF + MOG^35–55^. Like GMCSF-MOG, GMCSF-NFM also exhibited antigen targeting activity in that GMCSF-NFM was more active than the combination of GMCSF + NFM^13–37^ (Fig. 1f). Neither MOG^35–55^ nor NFM^13–37^ peptides had activity in the absence of GM-CSF (Fig. 1e, f). Notably, GMCSF-MOG resulted in more persistent antigen retention compared to GMCSF-NFM in these DC cultures, as measured by potency (i.e., left-shifted response curve) or magnitude of the antigenic response (i.e., note difference in *y*-axis scales). These data are consistent with the concept that MOG^35–55^/I-A^b^ complexes may be more durable than NFM^13–37^/I-A^b^ complexes [32]. These data indicate that antigen targeting is an attribute of the GM-CSF domain, which binds GM-CSF receptors on DCs to target and sequester covalently tethered antigens into the MHCII antigen processing compartment for subsequent presentation.

To assess the relative strengths of the antigenic peptides in an alternative assay, 2D2-FIG splenocytes were stimulated with MOG^35–55^ or NFM^13–37^ to measure the time-dependent accumulation of IL-2 in culture supernatants (Fig. 1g). At indicated time points (*x*-axis), supernatants from triplicate cultures were harvested and assayed for IL-2 activity by use of an IL-2 indicator cell line. As predicted, the high-efficiency NFM^13–37^ peptide resulted in significant IL-2 production within 1 day of culture, and IL-2 levels gradually attenuated until the end of the observation period at day 4. MOG^35–55^ also stimulated IL-2 production, but the response was delayed and was marked by low accumulations followed by rapid disappearance of IL-2 activity (Fig. 1g). Thus, even though GMSF-MOG and MOG did not induce CD40L expression, both antigens activated T cells in antigenic-targeting and IL-2 production assays, respectively.

### The high-efficiency GMCSF-NFM vaccine induced memory Tcon responses in 2D2-FIG mice

**Fig. 2:**
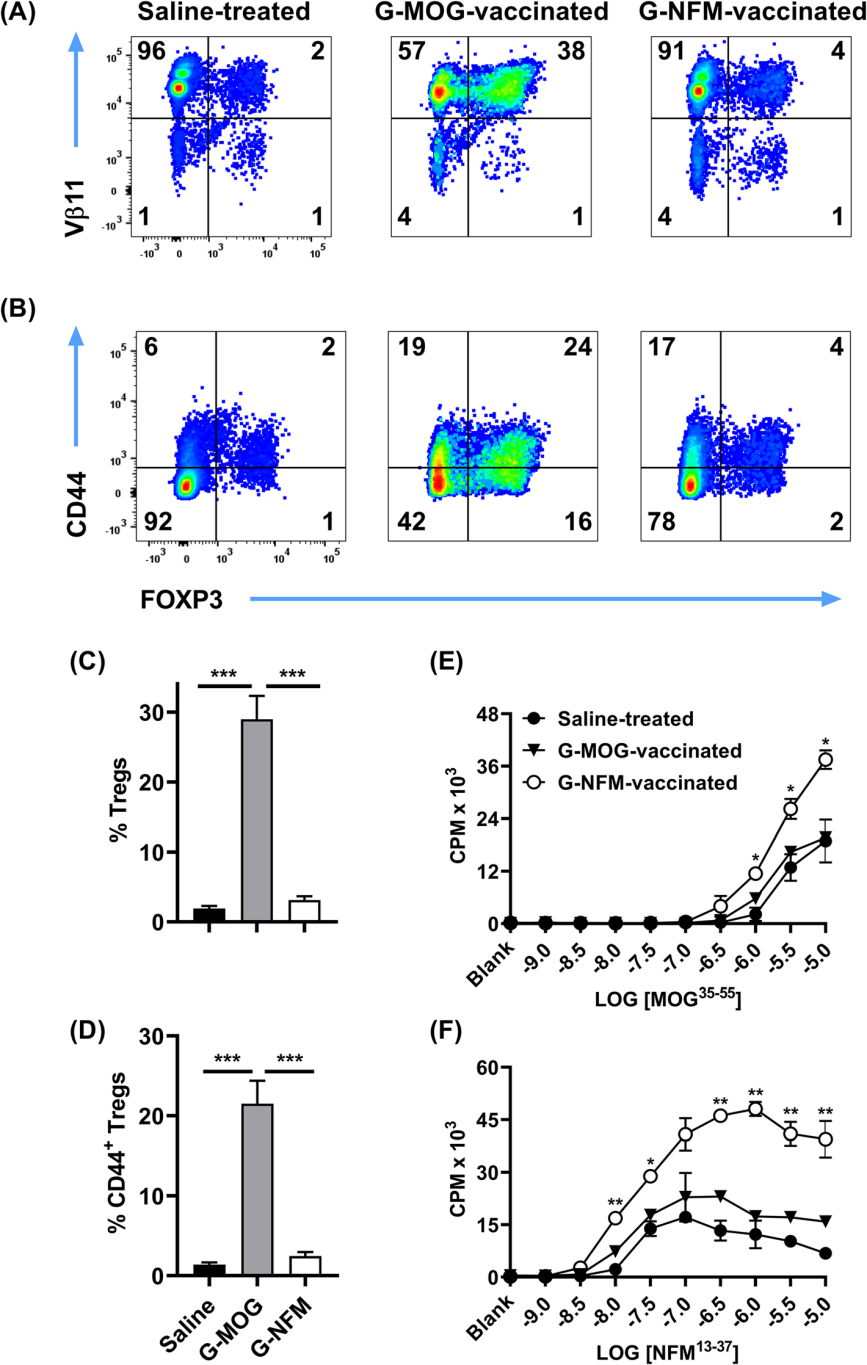
The high-efficiency GMCSF-NFM vaccine induced memory Tcon responses in 2D2-FIG mice. On day 0, 2D2-FIG mice were SC injected with 4 nmol of GMCSF-MOG, 4 nmol of GMCSF-NFM, or saline. On day 8, splenocytes were harvested from vaccinated mice, and CD4^+^ T cells were purified and analyzed for **a** Vβ11 (*y*-axis), **b** CD44 (*y*-axis), and **a**, **b** FOXP3 expression (*x*-axis). To assess generality of these findings (**c**, **d**), data from seven controlled experiments (analysis of PBMC ranging from day 4 to day 7) were pooled to assess 2D2-FIG mice vaccinated SC with saline (*n* = 24), 4 nmol of GMCSF-MOG (*n* = 24), or 4 nmol of GMCSF-NFM (*n* = 25) for total Treg percentages (**c**) and CD44^+^ Treg percentages (**d**) among CD4^+^ T cells. **a**, **b**, **e**, **f** Purified 2D2-FIG splenic T cells (25,000/well) from each vaccinated mouse were cultured in duplicate with 200,000 irradiated naïve splenocytes (C57BL/6) and designated concentrations (x-axis) of **e** MOG^35–55^ and **f** NFM^13–37^. Cultures were pulsed with 1 μCi of [^3^H]thymidine during the last 24 h of a 3-day culture. These data are representative of two independent experiments. Statistical significance was analyzed by use of a one-way ANOVA. (*p < 0.05, **p < 0.01, ***p < 0.001). Error bars represent standard deviations

Because GMCSF-NFM did not elicit Tregs in 2D2-FIG mice, we asked whether GMCSF-NFM instead elicited memory Tcon responses. To address this possibility, 2D2-FIG mice were vaccinated with 4 nmol of GMCSF-NFM, GMCSF-MOG, or saline, and splenocytes were analyzed 8 days post-vaccination for CD3, CD4, FOXP3, Vβ11 (2D2 TCRβ), and CD44 expression (Fig. 2). As expected, vaccination with GMCSF-NFM or saline resulted in baseline percentages (~ 1–4%) of Tregs whereas GMCSF-MOG elicited high percentages (~ 38%) of splenic CD4^+^ Vβ11^+^ Tregs (Fig. 2a). Interestingly, GMCSF-MOG elicited higher percentages of CD44^high^ T cells compared to GMCSF-NFM in both the Treg and Tcon compartments (Fig. 2b). In GMCSF-MOG vaccinated mice, 43% of splenic CD4^+^ T cells were CD44^high^ compared to 21% in GMCSF-NFM vaccinated 2D2-FIG mice. These data were consistent with experiments showing that GMCSF-MOG elicited significantly higher Treg percentages in PBMCs than GMCSF-NFM (~ 29% compared to ~ 3%) (Fig. 2c) together with significantly higher CD44^+^ Treg percentages (~ 22% compared to ~ 2%) (Fig. 2d). Antigen-specific recall responses were measured on day 8 post-vaccination by culturing purified, vaccine-induced CD4^+^ T cells with naïve irradiated splenic APC. T cells from GMCSF-NFM vaccinated mice had increased proliferative responses when re-stimulated with 1 μM–10 μM of MOG^35–55^ or 320 nM–10 μM NFM^13–37^ compared to T cells derived from GMCSF-MOG vaccinated or saline treated mice (Fig. 2e, f). GMCSF-MOG vaccinated mice had 2.5-fold more CD44^high^ T cells compared to GMCSF-NFM vaccinated mice yet exhibited T cell responses that resembled those of naïve T cells from the saline-treated mice. The reduced proliferative responsiveness in GMCSF-MOG vaccinated mice was likely due to the increased percentages of immunosuppressive GMCSF-MOG-induced Tregs (Fig. 2a). These results showed that the GMCSF-NFM vaccine induces a strong memory T cell response unimpeded by Tregs.

### Administration of a CD40 agonist in vivo inhibited the Treg-inductive activity of GMCSF-MOG

**Fig. 3:**
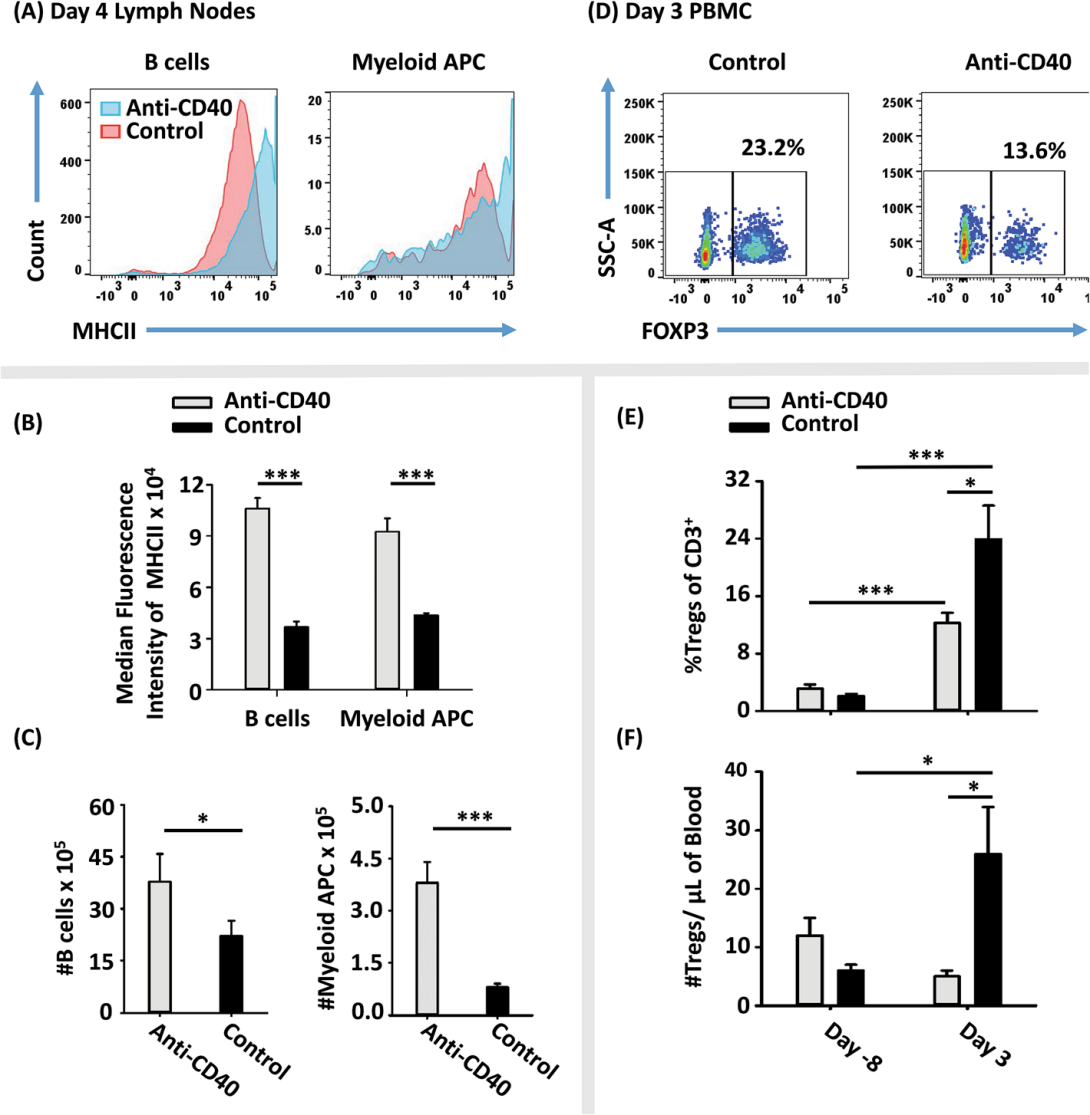
Administration of a CD40 agonist in vivo inhibited the Treg-inductive activity of GMCSF-MOG. On day − 2 and 0, 2D2-FIG mice (*n* = 7/group) were injected i.p. with 100 μg of an anti-CD40 mAb (clone FGK4.5, rat anti-mouse CD40, IgG2a) or control mAb (clone 2A2, rat anti-trinitrophenol, IgG2a) in 500 μl saline. All mice were injected with 4 nmol of GMCSF-MOG on day 0. PBMC were analyzed on day − 8 before vaccination and day 3 post-vaccination for side-scatter (SSC), CD3, and FOXP3. Lymph nodes were harvested and analyzed on day 4 for CD4, CD11b, MHCII, and FOXP3. Shown are **a** representative histograms analyzed for MHCII expression (*x*-axis), **b** MHCII median fluorescence intensity, and **c** numbers of CD4^−^ CD11b^−^ MHCII^+^ cells (B cells) and CD11b^+^ CD4^−^ cells (myeloid APC) from lymph nodes on day 4. Also shown are **d** representative dot plots of CD3^+^ T cells analyzed for SSC (*y*-axis) and FOXP3 (*x*-axis) from blood on day 3 together with percentages **e** and numbers **f** of Tregs (per μl of blood) on days − 8 and 3. Statistical significance was analyzed by use of a one-tailed *t* test. (*p < 0.05, ***p < 0.001). These data are representative of two independent experiments. Error bars represent SEM

Given the association of TCR recognition efficiency and CD40L induction, a central question was whether the independent activation of the CD40L/CD40 pathway would reverse the tolerogenic activity of GMCSF-MOG. To test this possibility, an agonistic anti-CD40 mAb was used to pre-activate APCs in vivo (Fig. 3). The anti-CD40 agonist (FGK4.5) or a control mAb (2A3) was injected (i.p. 100 μg) into 2D2-FIG mice on days − 2 and 0 followed by vaccination with GMCSF-MOG on day 0. PBMCs were analyzed on day 3 and lymph nodes were analyzed on day 4. As expected, anti-CD40 mAb treatment caused APC activation in vivo, as shown by increased median MHCII expression and elevated numbers of both CD11b^+^ cells and B cells compared to control mAb-treated mice (Fig. 3a–c). The anti-CD40 agonist profoundly diminished the Treg-inductive activities of GMCSF-MOG, in that anti-CD40-treated mice had significantly lower percentages of Tregs and fewer Tregs per μl of blood than mice treated with the control mAb (Fig. 3d–f). Indeed, the anti-CD40 agonist reduced numbers of Tregs in GMCSF-MOG-vaccinated mice to baseline levels of non-vaccinated mice (Fig. 3f). These data indicated that activation of CD40L/CD40 pathway counteracts the Treg-inductive activity of GMCSF-MOG.

### GMCSF-MOG induced robust Treg responses even when mixed with an immunogenic vaccine

**Fig. 4:**
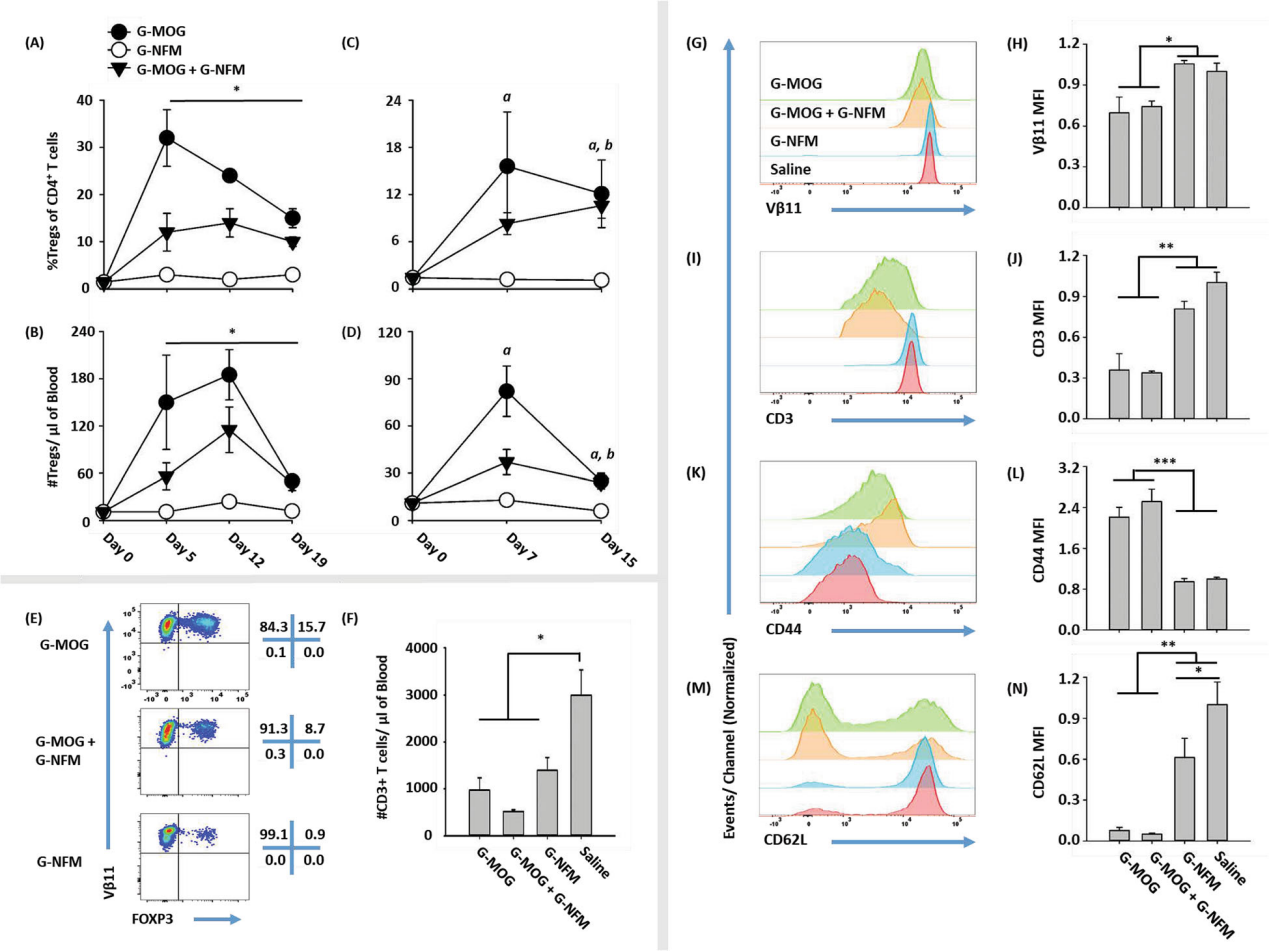
GMCSF-MOG induced robust Treg responses even when mixed with an immunogenic vaccine. **a**–**n** On day 0, 2D2-FIG (*n* = 3–5) mice were injected with 4 nmol of GMCSF-MOG, 4 nmol of GMCSF-NFM, or saline. Separate groups were also injected with either 2 nmol of GMCSF-MOG + 2 nmol of GMCSF-NFM (**a**, **b**) or 4 nmol of GMCSF-MOG + 4 nmol GMCSF-NFM (**c–n**). PBMCs were assayed for CD3, CD4, FOXP3, Vβ11 (2D2 TCRβ), CD44, and CD62L expression. Shown are **a**, **c** percentages and **b**, **d** numbers (per microliter of blood) of FOXP3^+^ Tregs for CD3^+^ CD4^+^ T cells collected on days 0, 5, 12, and 19 (**a**, **b**) or days 0, 7, and 15 (**c**, **d**). Also shown for day 7 are **e** representative dot plots of CD3^+^ CD4^+^ T cells analyzed for Vβ11 (*y*-axis) and FOXP3 expression (*x*-axis); **f** the total number of CD3^+^ T cells per μl of blood; representative histograms for **g** Vβ11, **i** CD3, **k** CD44, and **m** CD62L expression of CD3^+^ CD4^+^ T cells; and the mean florescence intensity (MFI) of **h** Vβ11, **j** CD3, **l** CD44, and **n** CD62L. **a**, **b** Statistical significance was analyzed by use of a two-way repeated measures ANOVA. Means for groups G-MOG, “G-MOG + G-NFM”, and G-NFM represented statistically significant differences compared to each other (*p < 0.05). **c**–**n** Statistical significance was analyzed by use of a one-way ANOVA; **c**, **d** (*a*) G-MOG vs G-NFM (*p < 0.05*) and (*b*) “G-MOG + G-NFM” vs G-NFM (p < 0.05). (*p < 0.05, **p < 0.01, ***p < 0.001). Error bars represent SEM

Given the expectation that tolerance requires a quiescent steady-state environment, the standard prediction is that weak tolerogenic interactions would be subdominant to strong immunogenic interactions, which would dominate to drive inflammation. However, previous studies showed that the tolerogenic [20] and Treg-inductive activities [21] of GMCSF-MOG were dominant even in lymphatics conditioned by CFA. Thus, a central question is how integration of disparate antigen recognition events determines T cell lineage fate when low and high-efficiency antigens are concurrently presented in the same draining lymphatic to the same clonotype. To address this question, the low-efficiency GMCSF-MOG vaccine was mixed with the high-efficiency GMCSF-NFM vaccine, and the combined vaccine was injected SC into 2D2-FIG mice. The vaccine dose was controlled by administering 2 nmol GMCSF-MOG + 2 nmol GMCSF-NFM (Fig. 4a, b) for a total of 4 nmol antigen or by administering 4 nmol of GMCSF-MOG + 4 nmol GMCSF-NFM (Fig. 4c–n) for a total of 8 nmol antigen. Control vaccine responses were assessed by vaccinating 2D2-FIG mice with 4 nmol of GMCSF-MOG, 4 nmol of GMCSF-NFM, or saline. Consistent with previous results, GMCSF-MOG alone elicited robust Treg responses in 2D2-FIG mice that were optimal 1–2 weeks post-vaccination and that gradually waned during the 3rd week whereas GMCSF-NFM, when administered alone, did not elicit Treg expansion and instead resulted in Treg percentages comparable to pre-vaccination levels (Fig. 4a, c). The mix of GMCSF-MOG + GMCSF-NFM resulted in intermediate but robust Treg responses from days 5–12 as measured by percentages or absolute numbers of Tregs/μl of blood (Fig. 4a–d). Representative dotplots are shown in Fig. 4e. These data showed that inefficient and efficient TCR interactions were integrated to provide balanced outcomes. These data did not support the standard assumption that efficient TCR interactions have immunodominance over inefficient TCR interactions.

GMCSF-MOG, GMCSF-NFM, and GMCSF-MOG + GMCSF-NFM vaccination resulted in a 2–5-fold reduction in the total number of circulating CD3^+^ T cells per microliter of blood compared to saline-vaccinated mice (Fig. 4f). Thus, antigen stimulation resulted in the decreased circulation of 2D2 T cells in the blood in response to either low-efficiency or high-efficiency antigen recognition. GMCSF-MOG and “GMCSF-MOG + GMCSF-NFM”-treated mice had decreased Vβ11, CD3, and CD62L expression as well as increased CD44 expression on a per cell basis compared to GMCSF-NFM and saline-treated mice on day 7 (Fig. 4g–n). Therefore, GMCSF-MOG selectively downregulated Vβ11 and CD3 and induced a CD44^high^ CD62L^low^ phenotype in CD4^+^ T cells, which most likely reflected persistence of antigen-experienced memory T cells due to the longevity of the response. Not only did low-efficiency antigen recognition (i.e., GMCSF-MOG) exert partial dominance in eliciting Treg responses when co-administered with GMCSF-NFM, GMCSF-MOG also induced phenotypic changes including decreased Vβ11, CD3, and CD62L and increased CD44 expression that were dominant over responses induced by high-efficiency antigen recognition (i.e., GMCSF-NFM).

### GMCSF-MOG was superior to GMCSF-NFM for eliciting persistence of a CD44^high^ Tcon phenotype

**Fig. 5:**
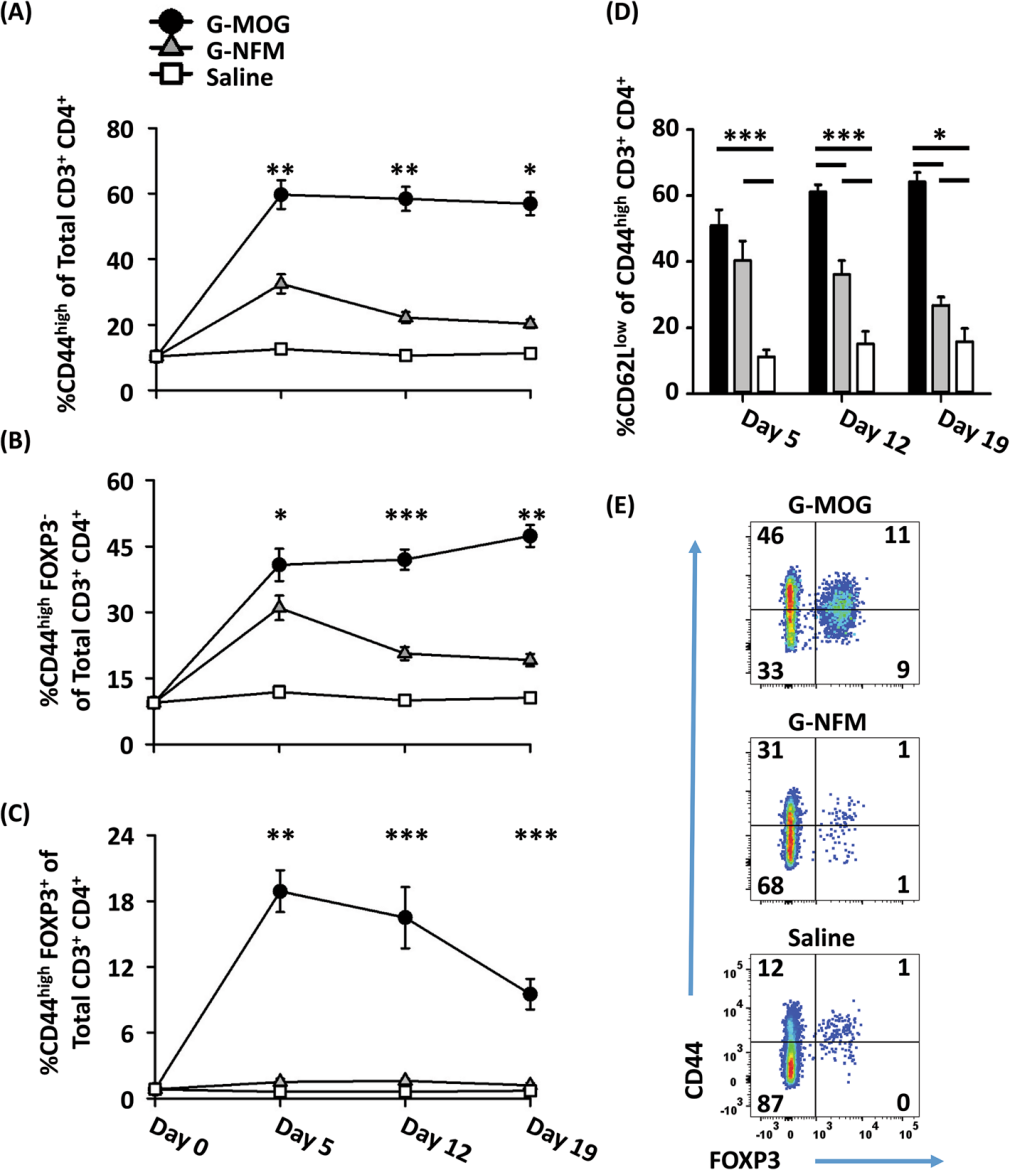
GMCSF-MOG was superior to GMCSF-NFM for eliciting persistence of a CD44^high^ Tcon phenotype. **a**–**d** 2D2-FIG mice were vaccinated with 4 nmol of GMCSF-MOG (*n* = 5), 4 nmol of GMCSF-NFM (*n* = 5), or with saline (*n* = 5). PBMCs were analyzed on days 5, 12, and 19 for CD3, CD4, CD44, and FOXP3 expression. **a**–**c** The day 0 time point was derived from the CD44 expression of CD3^+^ CD4^+^ T cells from naïve 2D2-FIG mice (*n* = 23). Shown are percentages of **a** CD44^high^ T cells, **b** CD44^high^ Tcons, and **c** CD44^high^ Tregs of gated CD3^+^ CD4^+^ T cells on days 0, 5, 12, and 19. Shown in **d** are the percentage of CD62L^low^ T cells of gated CD44^high^ CD3^+^ CD4^+^ T cells on day 5, 12, and 19. Shown in **e** are representative dot plots of CD44 (*y*-axis) and FOXP3 (*x*-axis) expression of CD4^+^ CD3^+^ T cells on day 5. These data are representative of three independent experiments. Statistical significance was analyzed using a one-way ANOVA. Shown in **a**, **b** and **d** are significant differences between the groups GMCSF-MOG, GMCSF-NFM, and saline, and **c** statistical differences comparing GMCSF-MOG treatment to both GMCSF-NFM and saline. (*p < 0.05, **p < 0.01, ***p < 0.001). **d** Asterisks apply to all comparisons at a given timepoint. Error bars represent SEM

The finding that GMCSF-MOG induced persistence of CD44^high^ memory T cells at levels higher than those induced by GMCSF-NFM (Fig. 4k, l) was paradoxical because GMCSF-NFM induction of the CD40L/CD40 co-stimulatory pathway would predictably be a more efficient driver of T cell activation and memory. To assess persistence of the CD44^high^ phenotype, 2D2-FIG mice were vaccinated with 4 nmol of GMCSF-MOG, GMCSF-NFM, or with saline. On days 5, 12, and 19, PBMCs were analyzed for percentages of CD44^high^ T cells (Fig. 5a, e), Tcons (Fig. 5b, e), and FOXP3^+^ Tregs (Fig. 5c, e) in the CD4^+^ T cell pool. The CD44 expression baseline on day 0 was derived from the CD44 expression of CD3^+^ CD4^+^ T cells from naïve 2D2-FIG mice (Fig. 5a–c). GMCSF-MOG elicited high percentages of CD44^high^ T cells such that ~ 55–60% of CD4^+^ T cells expressed CD44 from days 5–19 (Fig. 5a), which reflected increased percentages of both CD44^high^ Tcons (40–48%; Fig. 5b) and CD44^high^ Tregs (10–19%; Fig. 5c). Conversely, GMCSF-NFM elicited more moderate percentages of CD44^high^ T cells such that ~ 20–31% of CD4^+^ T cells expressed CD44 from days 5–19, which predominately reflected increased percentages of Tcons rather than Tregs (Fig. 5a–c). The GMCSF-MOG vaccine was not only associated with increased percentages of CD44^high^ T cells but also with increased percentages of memory T cells such that 51–64% of the CD44^high^ CD4^+^ T cells were CD62L^low^ from days 12–19 (Fig. 5d). Evidence for the memory status of these CD44^high^ T cells derives from the longevity of the response, which extended over the course of 19 days without any indication of attenuation. Conversely, GMCSF-NFM elicited lower percentages of CD44^high^ T cells that waned over time such that 40% of the CD44^high^ CD4^+^ T cells were CD62L^low^ on day 5 and waned to 26% by day 19 (Fig. 5d). These data confirmed that GMCSF-MOG vaccination elicits higher percentages of CD44^high^ memory T cells in both the Tcon and Treg pools of CD4^+^ T cells.

### The tolerogenic activity of GMCSF-MOG was associated with levels of pre-existing 2D2 Tregs

**Fig. 6:**
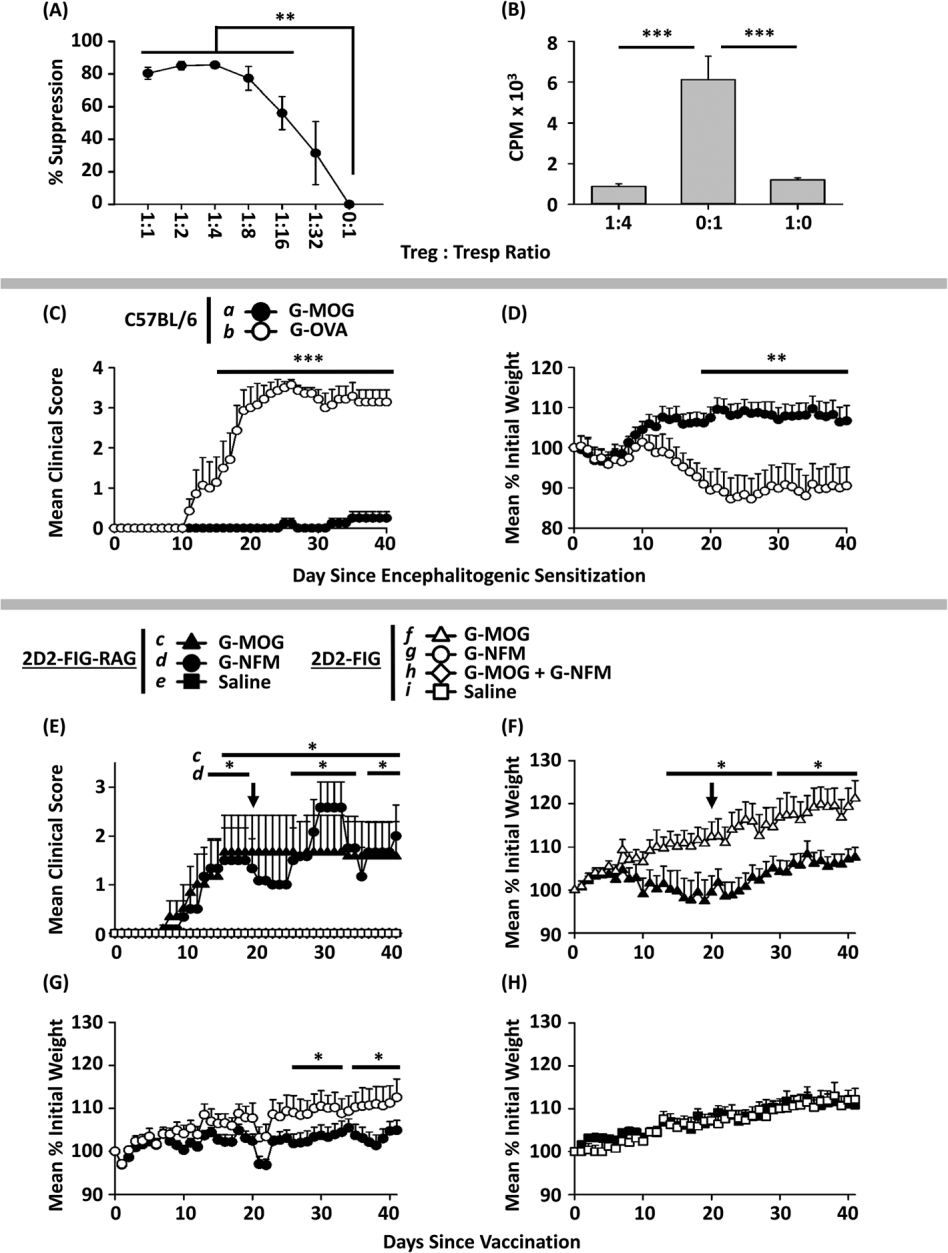
The tolerogenic activity of GMCSF-MOG was reflected by levels of pre-existing 2D2 Tregs. **a**, **b** 2D2-FIG mice were vaccinated with 4 nmol of GMCSF-MOG, and splenic Tregs were purified by flow sorting on day 7. Tregs were added to triplicate cultures of purified naïve splenic 2D2-FIG T responder cells that were supplemented with 10^5^ irradiated C57BL/6 splenocytes and 1 μM MOG^35–55^ as designated. Cultures were pulsed with 1 μCi of [^3^H]thymidine during the last 24 h of a 3-day culture. Shown in **a** are the percent suppression of maximal MOG-stimulated proliferation and **b** the counts per minute (CPM) from cultures containing a 1:4 Treg/responder mixture (Treg: Tresp ratio) versus Tregs alone versus T cell responders alone. **c**, **d** C57BL/6 mice were treated with 2 nmol GMCSF-MOG (*n* = 8) or GMCSF-OVA (*n* = 7) on days − 21, − 14, and − 7. On day 0, all mice were immunized with 200 μg MOG^35–55^ in CFA and 400 ng of Pertussis toxin i.p. on days 0 and 2. Shown in **c** are the daily mean clinical EAE scores and **d** the normalized mean body weight through the end of the experiment on day 40. **e**–**h** 2D2-FIG or 2D2-FIG-*Rag1*^*−/−*^ mice were treated with 4 nmol GMCSF-MOG, GMCSF-NFM, “GMCSF-MOG + GMCSF-NFM” (SC in saline), or with saline on days 0 and day 20 (arrow). Shown in **e** are the daily mean clinical EAE scores. Note that all open symbols (4 groups) and closed-square symbols (1 group) are included in the graph and are overlapping, reflecting no disease from days 0–41. Also shown in **f, g,** and **h** are the mean normalized body weight through the end of the experiment on day 41. **a**, **b** Statistical significance was analyzed using a two-tailed *t* test. Error bars represent standard deviation. **c**–**h** Differences between groups were analyzed using a two-way repeated measures ANOVA. Error bars represent SEM. **c**–**h** Significant differences between groups were **c**, **d** (*a*) vs (*b*); **e** (*c*) vs (*e–i*) and (*d*) vs (e*-i*); **f** (*c*) vs (*f*); and **g** (*d*) vs (*g*). **c**, **e** See Table 1 for clinical signs of EAE. These data are representative of (**c**–**h)** three or (**a**, **b**) two independent experiments. (*p < 0.05, **p < 0.01, ***p < 0.001)

Given that GMCSF-MOG induced both Tregs and resistance to EAE, a central question was whether GMCSF-MOG-induced Tregs had canonical suppressive activity. To assess this question, 2D2-FIG mice were vaccinated with 4 nmol of GMCSF-MOG to induce Tregs, which were purified by FACS on day 7. These MOG-specific Tregs were tested for suppressive activity at various ratios with purified naïve CD4^+^ 2D2-FIG T cell responders in the presence of irradiated splenic APCs and 1 μM MOG^35–55^ (Fig. 6a, b). These GMCSF-MOG-induced Tregs suppressed MOG-specific proliferative responses at Treg/T cell responder ratios equal to or above 1:16.

Previous research showed that GMCSF-MOG elicited delayed Treg responses in 2D2-FIG-*Rag1*^*−/−*^ mice which have reduced levels of pre-existing Tregs (~ 0.001–0.01% Tregs) compared to 2D2-FIG mice ( 0.5–2.0% Tregs) [21]. The delayed Treg response to the vaccine was attributed to a model in which GMCSF-MOG vaccination expanded pre-existing Tregs and thus required more time to mount a Treg response in 2D2-FIG-*Rag1*^*−/−*^ mice due to the minute starting Treg populations. Here, we investigated whether pre-existing Tregs were required for the induction of antigen-specific tolerance with GMCSF-MOG. Thus, GMCSF-MOG tolerogenic vaccination was assessed in the context of different Treg repertoires by comparing 2D2-FIG-*Rag1*^*−/−*^ mice (virtually devoid of Tregs), 2D2-FIG mice (a constrained MOG-specific Treg repertoire), and C57BL/6 mice (a fully replete Treg repertoire).

As shown previously [18], GMCSF-MOG vaccination prevented induction of EAE in C57BL/6 mice which have normal Treg frequencies and a wild type T cell repertoire. C57BL/6 mice were pretreated on days − 21, − 14, and − 7 with 2 nmol of either GMCSF-MOG (*n* = 8) or GMCSF-OVA (*n* = 7) and subsequently challenged with 200 μg MOG^35–55^ in CFA on day 0 and treated with 400 ng of Pertussis toxin on days 0 and 2 (Fig. 6c, d, Table 1). Pretreatment with GMCSF-MOG SC in saline prevented the subsequent induction of EAE. Only 2 of 8 mice showed mild signs of EAE with a mean maximum score of 0.3 ± 0.2 whereas 7 of 7 mice treated with GMCSF-OVA exhibited severe EAE with a mean maximum score of 3.9 ± 0.1 (Fig. 6c, Table 1). GMCSF-MOG also prevented EAE-associated weight loss (Fig. 6d). These results showed that GMCSF-MOG was an effective pretreatment that blocked pathogenic responses in C57BL/6 mice that had a replete Treg repertoire.

**Table 1:** The tolerogenic activity of GMCSF-MOG was commensurate with levels of pre-existing Tregs

Exp.	Strain	Induction of EAE	Group	Treatment	Incidence of EAE	Mean cumulative score	Mean maximal score	% Maximal weight loss
1	C57BL/6	Active	*a*	GMCSF-MOG	2 of 8	2.1 ± 1.5	0.3 ± 0.2	− 0.8 ± 2.1
1	C57BL/6	Active	*b*	GMCSF-OVA	7 of 7	81.1 ± 6.9	3.9 ± 0.1	17.4 ± 4.1
2	2D2-FIG-*Rag1*^*−/−*^	Vaccine	*c*	GMCSF-MOG	3 of 6	49.0 ± 22.4	1.7 ± 0.8	6.4 ± 4.3
2	2D2-FIG-*Rag1*^*−/−*^	Vaccine	*d*	GMCSF-NFM	5 of 6	48.3 ± 16.9	2.6 ± 0.5	4.4 ± 1.6
2	2D2-FIG-*Rag1*^*−/−*^	Vaccine	*e*	Saline	0 of 5	0.0 ± 0.0	0.0 ± 0.0	− 2.7 ± 0.9
2	2D2-FIG	Vaccine	*f*	GMCSF-MOG	0 of 6	0.0 ± 0.0	0.0 ± 0.0	− 4.0 ± 2.8
2	2D2-FIG	Vaccine	*g*	GMCSF-NFM	0 of 6	0.0 ± 0.0	0.0 ± 0.0	− 2.4 ± 1.8
2	2D2-FIG	Vaccine	*h*	GMCSF-MOG + GMCSF-NFM	0 of 6	0.0 ± 0.0	0.0 ± 0.0	− 0.7 ± 2.2
2	2D2-FIG	Vaccine	*i*	Saline	0 of 6	0.0 ± 0.0	0.0 ± 0.0	− 1.6 ± 0.9

Experiment 1: C57BL/6 mice were treated with 2 nmol GMCSF-MOG (*n* = 8) or GMCSF-OVA (*n* = 7) on days − 21, − 14, and − 7. On day 0, all mice were immunized with 200 μg MOG^35–55^ in CFA and 400 ng of Pertussis toxin i.p. on days 0 and 2. The % maximum weight loss between days 1 and 40 was analyzed with a two-tailed Student’s *t* test. (*a*) versus (*b*), *(p ≤ 0.001).* Negative values reflect weight gain. Mean cumulative and maximal scores were analyzed with a Mann-Whitney *U* test (*a*) versus (*b*), (*p ≤ 0.001*)

Experiment 2: 2D2-FIG (*n* = 6) or 2D2-FIG-*Rag1*^*−/−*^ (*n* = 5–6) mice were vaccinated with 4 nmol GMCSF-MOG, GMCSF-NFM, “GMCSF-MOG + GMCSF-NFM”, or with saline on days 0 and day 20. The mean cumulative and maximal scores were analyzed with a Kruskal-Wallis Test and adjusted by the Bonferroni correction. Mean cumulative scores (*d*) versus (*e*–*i*), (*p < 0.01*) and mean maximal scores (*c*) versus (*f*–*i*) (*p < 0.05*) and (*d*) versus (*e*–*i*), (*p < 0.01*)

An important question was whether GMCSF-MOG exhibited tolerogenic activity in Treg-deficient 2D2-FIG-*Rag1*^*−/−*^ mice. To address this question, Treg-deficient 2D2-FIG-*Rag1*^*−/−*^ and Treg-sufficient 2D2-FIG mice were vaccinated on days 0 and 20 with 4 nmol of GMCSF-MOG, GMCSF-NFM, or the combination of GMCSF-MOG + GMCSF-NFM (SC in saline) and were assessed for clinical EAE through day 41 (Fig. 6e, Table 1). No immunizing adjuvants such as CFA or Pertussis toxin were used in these experiments. The pre-existing Treg repertoire appeared to be a critical variable, because both GMCSF-MOG and GMCSF-NFM elicited chronic, non-resolving EAE and disease-associated weight loss in 2D2-FIG-*Rag1*^*−/−*^ mice (Fig. 6e–g) with an incidence of 3/6 and 5/6, respectively (Table 1). Notably, 2D2-FIG-*Rag1*^*−/−*^ mice that were treated with saline did not exhibit clinical signs of EAE or weight loss (Fig. 6e, h). Conversely, GMCSF-MOG, GMCSF-NFM, and “GMCSF-NFM + GMCSF-MOG” did not elicit EAE or weight loss in Treg-sufficient 2D2-FIG mice (Fig. 6 e–h). These data provided evidence that GMCSF-NAg tolerogenic vaccination requires a competent pre-existing Treg repertoire to confer resistance to EAE. This interpretation is consistent with the observation that in vivo depletion of Tregs abrogated vaccine-mediated tolerance in EAE [21]. These studies however cannot exclude the possibility that B cells or endogenous TCRα gene products that are absent in 2D2-FIG-*Rag1*^*−/−*^ mice but present in 2D2-FIG mice may contribute to GMCSF-MOG-induced Treg expansion in 2D2-FIG mice. One should note though that GMCSF-MOG efficiently elicited tolerance and inhibited EAE in B cell deficient mice [20].

### GMCSF-MOG preferentially expanded Tregs from a memory T cell pool

**Fig. 7:**
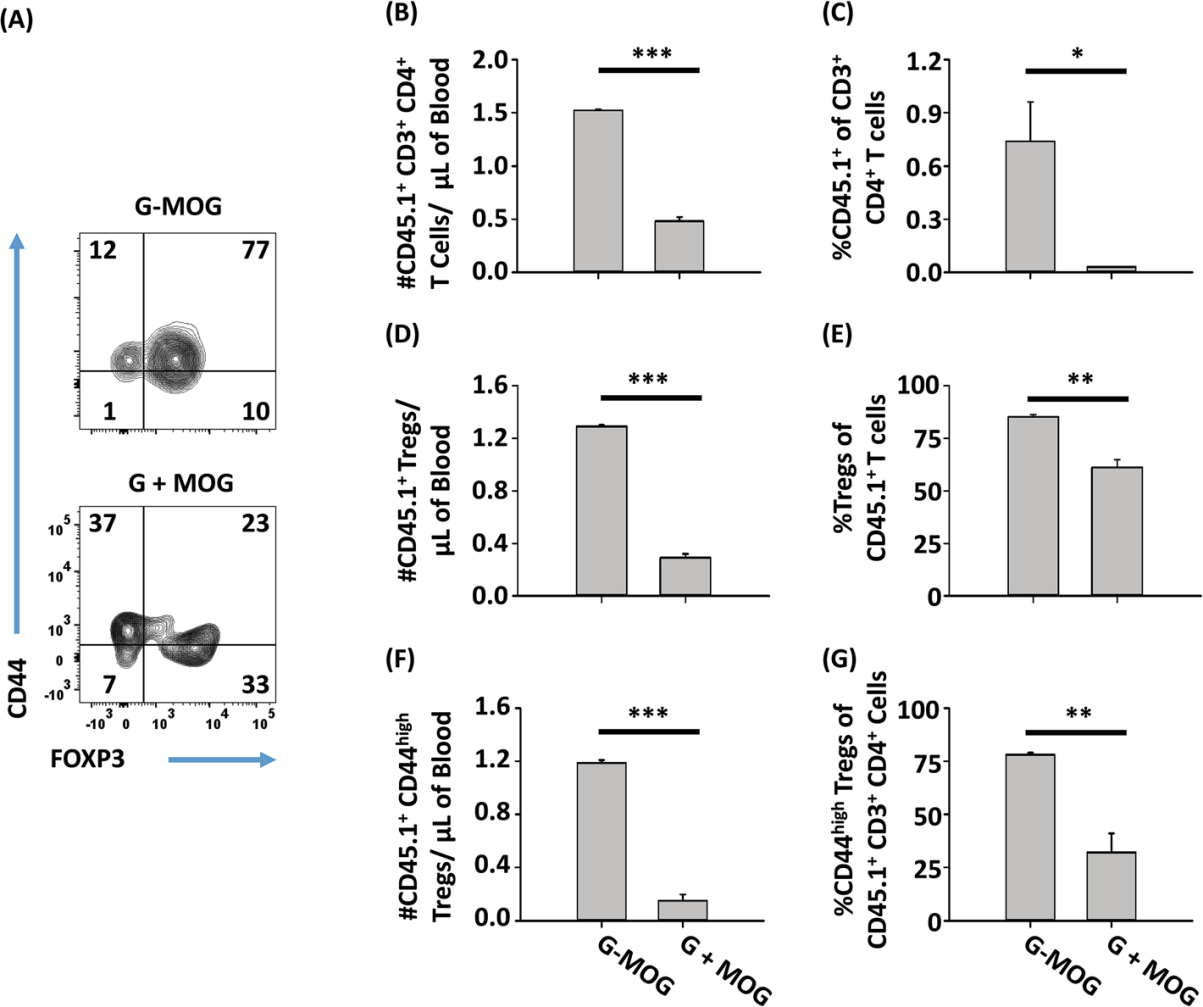
GMCSF-MOG preferentially expanded Tregs from a memory T cell pool. On day − 1, a CD45.1-2D2-FIG mixed Treg and Tcon line (40% Tregs and 60% Tcons) was injected intravenously (1.25 × 10^6^ cells) into CD45.2 2D2-FIG-*Rag1*^−/−^ mice (*n* = 3). On day 0, 2D2-FIG-*Rag1*^−/−^ recipient mice were vaccinated with 4 nmol of GMCSF-MOG or 4 nmol GM-CSF + 4 nmol MOG^35–55^. PBMCs were analyzed on day 4 for CD3, CD4, CD45.1, CD44, and FOXP3. Shown are analyses of donor CD45.1 CD3^+^ CD4^+^ T cells including **a** representative dot plots of GMCSF-MOG and “GM-CSF + MOG”-treated mice for CD44 (*y*-axis) and FOXP3 expression (*x*-axis) together with (**b**, **d**, **f**) cell numbers per microliter of blood and (**c**, **e**, **g**) percentages of designated cell populations. Statistical significance was analyzed by use of a two-tailed *t* test. (*p < 0.05, **p < 0.01, ***p < 0.001). Error bars represent SEM

A mixed CD45.1 2D2-FIG Treg and Tcon line (40% Tregs and 60% Tcons) was derived to provide independent verification that GMCSF-MOG drove clonal expansion of Tregs from pre-existing Treg pools. To generate these lines, FACS-sorted splenic Tregs or Tcons from CD45.1 2D2-FIG mice (4 × 10^5^/ml) were activated for 3 days with 2.5 μg/ml Con-A, IL-2, and irradiated DCs (10^5^/ml) in the presence or absence of 1 nM TGF-β, respectively. The Treg and Tcon lines were then propagated with IL-2, and Tregs were also cultured for 6 days with the anti-CD25 mAb PC61 to maintain Treg stability. After 13 days of culture, Tregs and Tcons were mixed and were transferred intravenously into CD45.2 2D2-FIG-*Rag1*^*−/−*^ hosts which were subsequently vaccinated one day later (on day 0) with 4 nmol of GMCSF-MOG or 4 nmol GM-CSF + 4 nmol MOG ^35–55^ (Fig. 7). Because donor T cells were expanded with Con-A and subsequently cultured for 13 days, donor T cells were considered an effector/memory subset. Among the adoptive T cell population, GMCSF-MOG vaccination resulted in increased percentages of CD44^high^ FOXP3^+^ Tregs compared to GM-CSF + MOG^35–55^ vaccinated mice (Fig. 7a). GMCSF-MOG increased the total adoptive Treg pool (Fig. 7d, e) and the CD44^+^ memory Treg pool (Fig. 7f, g) as assessed by circulating Treg numbers and percentages relative to the total CD4^+^ pool. Interestingly, GMCSF-MOG elicited a relative expansion of the total memory CD45.1 T cell population (~ 1.5 versus 0.5 CD45.1^+^ CD4^+^ T cells per microliter of blood) compared to GM-CSF + MOG^35–55^ treated 2D2-FIG-*Rag1*^*−/−*^ mice as measured by both numbers and percentages (Fig. 7b, c). These data provided evidence that GMCSF-MOG efficiently expanded CD44^+^ memory T cells coupled with a preferential expansion of CD44^+^ effector Tregs.

## Discussion

### GM-CSF has a tolerogenic dimension

In contrast to the widely held view that GM-CSF is primarily proinflammatory, there is mounting evidence that GM-CSF also has profound anti-inflammatory properties. For example, administration of exogenous GM-CSF reduces disease in numerous animal models of autoimmunity including myasthenia gravis, thyroiditis, type 1 diabetes, and graft versus host disease [33–37]. A consistent theme among these studies is that GM-CSF elicited the expansion of regulatory DC populations which, in turn, induced Tregs to reduce disease. In these studies, GM-CSF was administered without autoantigens. Thus, the effects of GM-CSF were antigen-independent or were dependent on endogenous auto-antigens in vivo. Because GM-CSF supports anti-inflammatory homeostatic processes, we suggest that GM-CSF amplifies the tolerogenic immune responses by supporting differentiation, function, and survival of regulatory myeloid DCs together with enhanced presentation of tissue-specific self-antigens to Tregs.

### The GMCSF-NAg model of tolerogenic vaccination

GMCSF-NAg fusion proteins are a vaccine platform that elicits robust NAg-specific tolerance. As such, this experimental system has implications for understanding basic mechanisms of Treg biology necessary to advance tolerogenic vaccination as a disease-specific intervention for autoimmune disease. The emerging model is that the GM-CSF vaccine domain binds to the GM-CSF receptor (CD116, CD131) on myeloid DCs to elicit receptor-mediated endocytosis thereby targeting the NAg vaccine domain to the MHCII antigen processing pathway for subsequent presentation of MHCII/NAg peptides. The GM-CSF domain signals through the GM-CSF receptor, which is postulated to enhance differentiation, survival, and function of myeloid DCs such that these GM-CSF-conditioned DCs are poised to present high concentrations of MHCII/NAg complexes. Due to the combined targeting and signaling activities of the GM-CSF domain, MHCII/NAg complexes derived from the NAg domain are presented by DCs to engage NAg-specific T cells and direct either tolerogenic or immunogenic responses based on TCR-MHCII/NAg recognition efficiency and CD40L expression levels.

We postulate that DC-mediated presentation of self-NAg predominately stimulates T cells below the threshold needed for CD40L expression and consequently expands NAg-specific FOXP3^+^ Tregs to mediate dominant tolerance. Presentation of self-antigens to CD4^+^ T cells are inherently low-efficiency antigen recognition events due to the antecedent thymic selection of the T cell repertoire and the elimination of clonotypes that recognize self-myelin antigens as high-efficiency ligands. Thus, most self-antigens elicit low-efficiency responses favoring Tregs and tolerance. In support, fusion proteins comprised of GM-CSF and other myelin epitopes induce tolerance and inhibit EAE [17–20]. Conversely, foreign antigens are not subject to thymic selection, and these nonself antigens are recognized as high-efficiency TCR ligands to elicit immunity. Thus, we postulate that foreign antigens presented by DCs to CD4^+^ T cells result in high-efficiency antigen recognition events that activate the CD40L/CD40 pathway to provide high levels of costimulation and pro-inflammatory cytokines that, in turn, drive Treg destabilization and robust induction of effector/memory Tcon responses. Consistent with this concept, vaccines comprised of GM-CSF and foreign viral or bacterial antigens have immunogenic pathogen-specific T cell activity [38–40].

### Vaccine-mediated tolerogenic activity was integrated via CD40L/CD40 pathway

Robust engagement of the TCR on CD4^+^ T cells results in CD40L expression which, in turn, engages CD40 to upregulate APC-mediated costimulation necessary for the feedback activation of antigen-engaged T-helper cells. CD40L thereby represents a major mechanism of T cell ‘help’ and APC ‘licensing’ by which macrophages, DCs, and B cells mediate adaptive cell-mediated and humor immunity [41]. Given the mechanistic relationship of CD40L expression with immunogenic responses, a critical question pertains to the functional role of TCR engagement in a window of TCR recognition efficiencies below those necessary for stimulation of CD40L expression.

This study provides two lines of evidence indicating that TCR recognition efficiencies below the CD40L-inductive threshold are formative for the antigenic expansion of the Treg niche. First, the TCR recognition efficiencies deficient or sufficient for induction of CD40L were associated with robust Treg or Tcon responses, respectively (Figs. 1 and 4). Second, in vivo administration of an anti-CD40 agonist inhibited the Treg-inductive activity of GMCSF-MOG (Fig. 3). These findings indicate that TCR recognition efficiency is integrated by expression of CD40L to determine the tolerogenic or immunogenic quality of the T cell response. Additional research will be needed to address how TCR recognition efficiencies are gauged by CD40L and other TNF-ligand superfamily members to regulate the relative balance of Tcon and Treg responses and thereby control outcomes of immunity or tolerance.

### Integration of diverse TCR signaling events by individual T cells

This study focuses on outcomes when low-efficiency and high-efficiency TCR ligands simultaneously impact the same T cell clonotype in the context of CNS tolerance and autoimmune disease. A central tenet of autoimmunity is based on the concept that pathogenic T cells in MS recognize self-CNS antigens as low-efficiency ligands, but pathogenicity is driven when the same clones recognize a foreign cross-reactive mimicry antigen as a high-efficiency driver of autoimmunity. Molecular mimicry is likely the rule rather than the exception, because mimicry is a common mechanism of immune evasion due to a strong evolutionary drive for infectious pathogens to mimic self-peptides as camouflage [42]. This raises several questions. First, why is infectious disease rarely rather than routinely accompanied by autoimmune disease? Second, why does tolerance to tissue-specific self-antigens dominate despite the concurrent exposure to strong foreign antigenic mimics of self during infectious disease?

The 2D2 model can be an informative system for investigating cross-reactive pathogenic T cell responses. In the 2D2 TCR model, MOG^35–55^ represents the prototypic low-efficiency self-antigen whereas NFM^13–37^ represents a fortuitous high-efficiency self-antigen that is cross-reactive with MOG^35–55^ [43]. NFM^13–37^ is not independently pathogenic in C57BL/6 mice, because NFM^13–37^ is likely recognized by clonotypically distinct NFM-specific Tregs that are not present in the constrained MOG-specific 2D2 model. The caveat is that 2D2 T cells have the capacity to mediate EAE upon recognition of either MOG or NFM in the CNS [43]. Although NFM^13–37^ is a self-antigen, the principles revealed in this study may nonetheless extrapolate to strongly agonistic antigens in general regardless of self or non-self-origins.

This study provides insight by showing that inefficient self-antigens elicit robust Treg expansion even in the presence efficacious cross-reactive antigens. The low-efficiency GMCSF-MOG vaccine lacked CD40L-inductive activity (Fig. 1), exhibited robust memory Treg-inductive activity within 3–4 days post-vaccination (Fig. 2), and mediated tolerance in EAE (Fig. 6). The functional relationship of low-efficiency TCR recognition and Treg expansion was also contingent upon the lack of CD40L signaling, because forced CD40L signaling inhibited Treg expansion (Fig. 3). In contrast, the high-efficiency GMCSF-NFM vaccine elicited CD40L in 2D2-FIG splenocytes (Fig. 1) and induced memory responses by antigen-experienced CD44^+^ Tcons (Fig. 2), which included CD44^+^ memory T cells and possibly other activated or effector CD44^+^ T cell subsets. GMCSF-NFM however was essentially devoid of Treg-inductive activity. Although one might anticipate that an immunogenic vaccine may be dominant, GMCSF-MOG elicited robust Treg responses even when mixed with GMCSF-NFM (Fig. 4). This finding indicated that the deficiency of GMCSF-MOG-induced CD40L expression was not a passive deficit but rather effectively counterbalanced the CD40L-inductive GMCSF-NFM vaccine (Fig. 4). When the low-efficiency GMCSF-MOG and high-efficiency GMCSF-NFM vaccines were given simultaneously, the outcome was intermediate for Treg percentages and numbers during the initial 1–2 weeks post-vaccination, whereas the two groups did not differ 15 days post-vaccination or thereafter as Tregs gradually disappeared from the circulation (Fig. 4).

### GMCSF-MOG profoundly affected the phenotype of the MOG-specific Tcon repertoire

The longevity of vaccine action of GMCSF-MOG compared to GMCSF-NFM was apparent not only for induction of FOXP3 expression but was also evident for CD44 expression in that GMCSF-MOG was superior compared to GMCSF-NFM in eliciting CD44^+^ Tcons during a 19-day observation period (Fig. 5). These data provided evidence that GMCSF-MOG, via superior persistence of the vaccine antigen or via a direct vaccine activity, elicited responses of superior duration compared to GMCSF-NFM. GMCSF-MOG was also dominant compared to GMCSF-NFM for induction of a desensitized T cell phenotype in that GMCSF-MOG caused the sustained downregulation of TCR expression (i.e., Vβ11 and CD3 expression) in addition to induction of an effector/memory phenotype (i.e., downregulation of CD62L and upregulation of CD44). GMCSF-MOG was dominant because this desensitized phenotype was evident in the presence or absence of GMCSF-NFM, whereas GMCSF-NFM lacked these activities (Fig. 4). The capacity of GMCSF-MOG to downregulate TCR expression (Fig. 4) and upregulate CD44 in Tcons persisted several weeks without attenuation (Fig. 5). These findings underscore the conclusion that GMCSF-MOG fundamentally altered both Treg and Tcon repertoires by mechanisms that were partially dominant (Tregs) or completely dominant (Tcons). The functional implications of GMCSF-MOG action on the Tcon repertoire are not understood, although Tcon desensitization may represent a synergistic mechanism of tolerance or anergy.

### Effective Treg responses were associated with a pre-existing Treg pool

Tolerogenic outcomes appeared to require an established Treg repertoire, which may be a foundational factor controlling outcomes when a T cell clone recognizes an inefficient self-antigen concurrently with a strong agonistic antigen (Fig. 6). Whether GMCSF-NAg induces de novo Treg differentiation is unknown, although several lines of evidence indicate that GMCSF-MOG drives clonal expansion of pre-existing MOG-specific Tregs. First, the kinetics of GMCSF-MOG-induced Treg responses were associated with starting frequencies of pre-existing Tregs. That is, GMCSF-MOG elicited robust Treg responses within 3–4 days in 2D2-FIG mice but required 9–10 days to elicit similar Treg responses in 2D2-FIG-*Rag1*^−/−^ mice, and these kinetics correlated closely with starting Treg frequencies (~ 1% versus ~ 0.01–0.1% circulating Tregs, respectively) [21]. Second, in CD45.2 2D2-FIG-*Rag1*^−/−^ mice bearing an adoptive mixed population of CD45.1-2D2-FIG Treg and Tcon cells, GMCSF-MOG caused selective CD45.1 Treg expansion within 3 days (Fig. 7). Third, GMCSF-NAg appeared to require pre-existing Tregs for the induction of tolerance because GMCSF-MOG induced EAE in 2D2-FIG-*Rag1*^−/−^ mice but not in 2D2-FIG mice (Fig. 6). Coupled with the observation that in vivo depletion of Tregs abrogates vaccine-mediated tolerogenic activity [21], these data provide evidence that GMCSF-NAg drives clonal expansion of pre-existing Tregs to elicit NAg-specific tolerance. Although 2D2-FIG-*Rag1*^−/−^ mice also lacked B cells and endogenous TCRα rearrangements, the profound deficit in Treg numbers represents the most direct explanation for disease susceptibility, due to the close functional relationship of Tregs with tolerance [14, 21]. This study thereby presents the prospect that susceptibility to autoimmune disease represents an unlikely coincidence of a mimicry-stimulated autoimmune Tcon response coupled with a tissue-specific deficiency in the Treg repertoire (i.e., a hole in the Treg repertoire) that allows unmitigated autoimmune pathogenesis to occur in a target tissue.

### The GMCSF-NAg vaccines elicited tolerogenic activity in inflammatory environments

Many tolerogenic vaccines, if rendered in a strong immunological adjuvant, would predictably elicit immunity rather than tolerance. For example, high doses of naked myelin peptides delivered intravenously or via other routes (e.g., oral, intranasal, skin patch) can be used to induce tolerance and provide protection against the subsequent induction of EAE [44, 45]. However, the same naked myelin peptides emulsified in CFA are known to cause EAE [46]. These observations have been used to argue that tolerogenic vaccines require quiescent steady-state environments to mediate tolerance. Indeed, many tolerogenic vaccine platforms may require resting steady-state conditions for induction of tolerance, but these systems cannot be used to extrapolate this concept as a universal generality. The conceptual incongruity is that if tolerance is preempted by inflammation, one would expect that infection and injury would routinely spiral to autoimmunity, which is not the case. The example of GMCSF-NAg tolerogenic vaccines challenges this steady-state hypothesis and instead supports the concept that tolerance can be efficiently induced and maintained even in proinflammatory environments. Several lines of evidence support the conclusion that GMCSF-NAg tolerogenic vaccines are operative in a proinflammatory environment. First, GMCSF-NAg effectively reversed established EAE as a therapeutic vaccine when administration was initiated after the onset of severe paralytic EAE [17, 18, 20]. Second, GMCSF-NAg was an effective tolerogen that inhibited the subsequent induction of EAE in wildtype and B cell-deficient mice even when the vaccine was mixed with CFA or administered in saline adjacent to MOG^35–55^/CFA encephalitogenic emulsion [20]. Third, when emulsified in CFA or Alum adjuvants, GMCSF-NAg induced FOXP3^+^ Treg responses that were robust, systemic, and rapid [21]. Fourth, this study shows that GMCSF-MOG elicited robust Treg responses when given simultaneously with the strongly agonistic vaccine GMCSF-NFM (Fig. 6). Overall, these data provide compelling evidence that GMCSF-NAg vaccines can impose tolerogenic outcomes in inflammatory environments, given the caveat that the tolerogenic vaccine most likely requires engagement by a pre-existing NAg-specific Treg repertoire.

## Conclusions

This study revealed a pathway by which inefficient TCR recognition of self-NAg/MHCII ligands at TCR signaling strengths below the CD40L inductive threshold was coupled with NAg-specific expansion of myelin-specific FOXP3^+^ Tregs and resistance to EAE. GM-CSF was used as a fusion partner to target low-efficiency myelin antigens to this tolerogenic pathway to drive Treg responses and inhibit EAE. Importantly, the tolerogenic vaccine GMCSF-MOG efficiently stimulates this homeostatic pathway to alleviate EAE by mechanisms that are operable in both quiescent and inflammatory environments.

## Data Availability

Data and data analyses of this study are largely included in this article. Complete datasets used and/or analyzed in the current study are available from the corresponding author upon reasonable request.

## Abbreviations

APC: Antigen presenting cell
CFA: Complete Freund’s adjuvant
CNS: Central nervous system
CPM: Counts per minute
DC: Dendritic cell
EAE: Experimental autoimmune encephalomyelitis
GM-CSF: Granulocyte-macrophage colony stimulating factor
i.p.: Intraperitoneal
mAb: Monoclonal antibody
MBP: Myelin basic protein
MFI: Mean fluorescence intensity
MHCII: major histocompatibility complex class II
MOG: Myelin oligodendrocyte glycoprotein
MS: Multiple sclerosis
NAg: Neuroantigen
NFM: Neurofilament medium
OVA: Ovalbumin
PBMC: Peripheral blood mononuclear cells
PBS: Phosphate-buffered saline
PLP: Proteolipid protein
SC: Subcutaneous or subcutaneously
SEM: Standard error of the mean
Tcon: T conventional cell
TCR: T cell receptor
Treg: Regulatory T cell

## References

[1] Dobson R, Giovannoni G (2019). Multiple sclerosis - a review. Eur J Neurol.

[2] Williamson EM, Berger JR (2015). Infection risk in patients on multiple sclerosis therapeutics. CNS Drugs.

[3] Ragonese P, Aridon P, Vazzoler G, Mazzola MA, Lo Re V, Lo Re M, Realmuto S, Alessi S, D'Amelio M, Savettieri G, Salemi G (2017). Association between multiple sclerosis, cancer risk, and immunosuppressant treatment: a cohort study. BMC Neurol.

[4] Claflin SB, Broadley S, Taylor BV (2018). The Effect of Disease Modifying Therapies on Disability Progression in Multiple Sclerosis: A Systematic Overview of Meta-Analyses. Front Neurol.

[5] Serra P, Santamaria P (2019). Antigen-specific therapeutic approaches for autoimmunity. Nat Biotechnol.

[6] Mannie MD, Curtis AD (2013). Tolerogenic vaccines for Multiple sclerosis. Hum Vaccin Immunother.

[7] Hohlfeld R, Dornmair K, Meinl E, Wekerle H (2016). The search for the target antigens of multiple sclerosis, part 1: autoreactive CD4+ T lymphocytes as pathogenic effectors and therapeutic targets. Lancet Neurol.

[8] Jones A, Hawiger D (2017). Peripherally Induced Regulatory T Cells: Recruited Protectors of the Central Nervous System against Autoimmune Neuroinflammation. Front Immunol.

[9] van der Vliet HJ, Nieuwenhuis EE (2007). IPEX as a result of mutations in FOXP3. Clin Dev Immunol.

[10] Ramsdell F, Ziegler SF (2014). FOXP3 and scurfy: how it all began. Nat Rev Immunol.

[11] Akirav EM, Bergman CM, Hill M, Ruddle NH (2009). Depletion of CD4(+)CD25(+) T cells exacerbates experimental autoimmune encephalomyelitis induced by mouse, but not rat, antigens. J Neurosci Res.

[12] Koutrolos M, Berer K, Kawakami N, Wekerle H, Krishnamoorthy G (2014). Treg cells mediate recovery from EAE by controlling effector T cell proliferation and motility in the CNS. Acta Neuropathol Commun.

[13] Ghosh D, Curtis AD, Wilkinson DS, Mannie MD (2016). Depletion of CD4+ CD25+ regulatory T cells confers susceptibility to experimental autoimmune encephalomyelitis (EAE) in GM-CSF-deficient Csf2-/- mice. J Leukoc Biol.

[14] Wilkinson DS, Ghosh D, Nickle RA, Moorman CD, Mannie MD. Partial CD25 antagonism enables dominance of antigen-inducible CD25high FOXP3+ regulatory T cells as a basis for a Treg-based adoptive immunotherapy. Front Immunol. 2017; 10.3389/fimmu.2017.01782. 10.3389/fimmu.2017.01782 PMC5735073 29312311

[15] Danikowski KM, Jayaraman S, Prabhakar BS (2017). Regulatory T cells in multiple sclerosis and myasthenia gravis. J Neuroinflammation.

[16] Dendrou CA, Fugger L, Friese MA (2015). Immunopathology of multiple sclerosis. Nat Rev Immunol.

[17] Blanchfield JL, Mannie MD (2010). A GMCSF-neuroantigen fusion protein is a potent tolerogen in experimental autoimmune encephalomyelitis (EAE) that is associated with efficient targeting of neuroantigen to APC. J Leukoc Biol.

[18] Abbott DJ, Blanchfield JL, Martinson DA, Russell SC, Taslim N, Curtis AD, Mannie MD (2011). Neuroantigen-specific, tolerogenic vaccines: GM-CSF is a fusion partner that facilitates tolerance rather than immunity to dominant self-epitopes of myelin in murine models of experimental autoimmune encephalomyelitis (EAE). BMC Immunol.

[19] Mannie MD, Blanchfield JL, Islam SM, Abbott DJ (2012). Cytokine-neuroantigen fusion proteins as a new class of tolerogenic, therapeutic vaccines for treatment of inflammatory demyelinating disease in rodent models of multiple sclerosis. Front Immunol.

[20] Islam SM, Curtis AD, Taslim N, Wilkinson DS, Mannie MD (2014). GM-CSF-neuroantigen fusion proteins reverse experimental autoimmune encephalomyelitis and mediate tolerogenic activity in adjuvant-primed environments: association with inflammation-dependent, inhibitory antigen presentation. J Immunol.

[21] Moorman CD, Curtis AD, Bastian AG, Elliott SE, Mannie MD (2018). A GMCSF-Neuroantigen Tolerogenic Vaccine Elicits Systemic Lymphocytosis of CD4(+) CD25(high) FOXP3(+) Regulatory T Cells in Myelin-Specific TCR Transgenic Mice Contingent Upon Low-Efficiency T Cell Antigen Receptor Recognition. Front Immunol.

[22] Yazdani R, Fekrvand S, Shahkarami S, Azizi G, Moazzami B, Abolhassani H, Aghamohammadi A (2019). The hyper IgM syndromes: Epidemiology, pathogenesis, clinical manifestations, diagnosis and management. Clin Immunol.

[23] Samoilova EB, Horton JL, Zhang H, Chen Y (1997). CD40L blockade prevents autoimmune encephalomyelitis and hampers TH1 but not TH2 pathway of T cell differentiation. J Mol Med (Berl).

[24] Laman JD, Maassen CB, Schellekens MM, Visser L, Kap M, de Jong E, van Puijenbroek M, van Stipdonk MJ, van Meurs M, Schwarzler C, Gunthert U (1998). Therapy with antibodies against CD40L (CD154) and CD44-variant isoforms reduces experimental autoimmune encephalomyelitis induced by a proteolipid protein peptide. Mult Scler.

[25] Howard LM, Miga AJ, Vanderlugt CL, Dal Canto MC, Laman JD, Noelle RJ, Miller SD (1999). Mechanisms of immunotherapeutic intervention by anti-CD40L (CD154) antibody in an animal model of multiple sclerosis. J Clin Invest.

[26] Girvin AM, Dal Canto MC, Miller SD (2002). CD40/CD40L interaction is essential for the induction of EAE in the absence of CD28-mediated co-stimulation. J Autoimmun.

[27] Aarts S, Seijkens TTP, van Dorst KJF, Dijkstra CD, Kooij G, Lutgens E (2017). The CD40-CD40L Dyad in Experimental Autoimmune Encephalomyelitis and Multiple Sclerosis. Front Immunol.

[28] Mannie MD, Fraser DJ, McConnell TJ (2003). IL-4 responsive CD4+ T cells specific for myelin basic protein: IL-2 confers a prolonged postactivation refractory phase. Immunol Cell Biol.

[29] Wilkinson DS, Ghosh D, Nickle RA, Moorman CD, Mannie MD (2017). Partial CD25 Antagonism Enables Dominance of Antigen-Inducible CD25(high) FOXP3(+) Regulatory T Cells As a Basis for a Regulatory T Cell-Based Adoptive Immunotherapy. Front Immunol.

[30] Abbas AK, Benoist C, Bluestone JA, Campbell DJ, Ghosh S, Hori S, Jiang S, Kuchroo VK, Mathis D, Roncarolo MG (2013). Regulatory T cells: recommendations to simplify the nomenclature. Nat Immunol.

[31] Karimi S, Chattopadhyay S, Chakraborty NG (2015). Manipulation of regulatory T cells and antigen-specific cytotoxic T lymphocyte-based tumour immunotherapy. Immunology.

[32] Lucca LE, Axisa PP, Aloulou M, Perals C, Ramadan A, Rufas P, Kyewski B, Derbinski J, Fazilleau N, Mars LT, Liblau RS (2016). Myelin oligodendrocyte glycoprotein induces incomplete tolerance of CD4(+) T cells specific for both a myelin and a neuronal self-antigen in mice. Eur J Immunol.

[33] Sheng JR, Li L, Ganesh BB, Vasu C, Prabhakar BS, Meriggioli MN (2006). Suppression of experimental autoimmune myasthenia gravis by granulocyte-macrophage colony-stimulating factor is associated with an expansion of FoxP3+ regulatory T cells. J Immunol.

[34] Hotta M, Yoshimura H, Satake A, Tsubokura Y, Ito T, Nomura S (2019). GM-CSF therapy inhibits chronic graft-versus-host disease via expansion of regulatory T cells. Eur J Immunol.

[35] Cheatem D, Ganesh BB, Gangi E, Vasu C, Prabhakar BS (2009). Modulation of dendritic cells using granulocyte-macrophage colony-stimulating factor (GM-CSF) delays type 1 diabetes by enhancing CD4+CD25+ regulatory T cell function. Clin Immunol.

[36] Ganesh BB, Cheatem DM, Sheng JR, Vasu C, Prabhakar BS (2009). GM-CSF-induced CD11c+CD8a--dendritic cells facilitate Foxp3+ and IL-10+ regulatory T cell expansion resulting in suppression of autoimmune thyroiditis. Int Immunol.

[37] Park MY, Lim BG, Kim SY, Sohn HJ, Kim S, Kim TG (2019). GM-CSF Promotes the Expansion and Differentiation of Cord Blood Myeloid-Derived Suppressor Cells. Which Attenuate Xenogeneic Graft-vs.-Host Disease. Front Immunol.

[38] Rodriguez D, Rodriguez JR, Llorente M, Vazquez I, Lucas P, Esteban M, Martinez AC, del Real G (1999). A human immunodeficiency virus type 1 Env-granulocyte-macrophage colony-stimulating factor fusion protein enhances the cellular immune response to Env in a vaccinia virus-based vaccine. J Gen Virol.

[39] Lu H, Xing Z, Brunham RC (2002). GM-CSF transgene-based adjuvant allows the establishment of protective mucosal immunity following vaccination with inactivated Chlamydia trachomatis. J Immunol.

[40] Li N, Yu YZ, Yu WY, Sun ZW (2011). Enhancement of the immunogenicity of DNA replicon vaccine of Clostridium botulinum neurotoxin serotype A by GM-CSF gene adjuvant. Immunopharmacol Immunotoxicol.

[41] Elgueta R, Benson MJ, de Vries VC, Wasiuk A, Guo Y, Noelle RJ (2009). Molecular mechanism and function of CD40/CD40L engagement in the immune system. Immunol Rev.

[42] Elde NC, Malik HS (2009). The evolutionary conundrum of pathogen mimicry. Nat Rev Microbiol.

[43] Krishnamoorthy G, Saxena A, Mars LT, Domingues HS, Mentele R, Ben-Nun A, Lassmann H, Dornmair K, Kurschus FC, Liblau RS, Wekerle H (2009). Myelin-specific T cells also recognize neuronal autoantigen in a transgenic mouse model of multiple sclerosis. Nat Med.

[44] Turley DM, Miller SD (2010). Prospects for antigen-specific tolerance based therapies for the treatment of multiple sclerosis. Results Probl Cell Differ.

[45] Badawi AH, Siahaan TJ (2012). Immune modulating peptides for the treatment and suppression of multiple sclerosis. Clin Immunol.

[46] Glatigny S, Bettelli E. Experimental Autoimmune Encephalomyelitis (EAE) as Animal Models of Multiple Sclerosis (MS). Cold Spring Harb Perspect Med. 2018;8. 10.1101/cshperspect.a028977 PMC6211376 29311122

